# Supplementary material for: Healthy lifestyle and life expectancy in people with multimorbidity in the UK Biobank: A longitudinal cohort study
Source: PLoS Med. 2020 Sep 22;17(9):e1003332. doi: 10.1371/journal.pmed.1003332 (PMC7508366; doi:10.1371/journal.pmed.1003332)
Supplement: S1 Text — (DOCX) [file pmed.1003332.s001.docx]

# **S1 Text:** List of the 36 chronic conditions included within the definition of multimorbidity

| 1. Anaemia |
| --- |
| 1. Angina |
| 1. Anxiety or panic attacks |
| 1. Asthma |
| 1. Atrial fibrillation |
| 1. Bronchiectasis |
| 1. Cancer |
| 1. Chronic kidney disease |
| 1. Chronic obstructive pulmonary disease (COPD) |
| 1. Chronic sinusitis |
| 1. Cirrhosis |
| 1. Dementia |
| 1. Depression |
| 1. Diabetes |
| 1. Eczema or dermatitis |
| 1. Epilepsy |
| 1. Glaucoma |
| 1. Heart failure |
| 1. Hepatitis |
| 1. Hypertension |
| 1. Inflammatory bowel disease |
| 1. Irritable bowel syndrome |
| 1. Meningitis |
| 1. Migraine |
| 1. Multiple sclerosis |
| 1. Myocardial infarction |
| 1. Osteoporosis |
| 1. Parkinson’s disease |
| 1. Peripheral vascular disease |
| 1. Prostate problem |
| 1. Rheumatoid arthritis |
| 1. Schizophrenia |
| 1. Stroke |
| 1. Thyroid problem |
| 1. Tuberculosis |
| 1. Vestibular disorder |
