# Supplementary material for: Healthy lifestyle and life expectancy in people with multimorbidity in the UK Biobank: A longitudinal cohort study
Source: PLoS Med. 2020 Sep 22;17(9):e1003332. doi: 10.1371/journal.pmed.1003332 (PMC7508366; doi:10.1371/journal.pmed.1003332)
Supplement: S2 Text — (DOCX) [file pmed.1003332.s002.docx]

# **S2 Text:** Weighted healthy lifestyle score

| Healthy lifestyle factor in the model | **β coefficient** | **Weighted**  **β coefficient** |
| --- | --- | --- |
| Regular physical activity (Yes vs No) | -0.2923622 | 0.204832 |
| No current smoking (Yes vs No) | -0.9015466 | 0.631632 |
| None/moderate alcohol consumption (Yes vs No) | -0.0798640 | 0.055953 |
| Healthy diet (Yes vs No) | -0.1535554 | 0.107582 |
| Total | -1.4273282 | 1 |

Regular physical activity: ≥500 MET-minutes/week.

None/moderate alcohol consumption: 0 to 14 units of alcohol a week.

Healthy diet: at least five portions of fruit and vegetables every day.

β coefficients have been estimated using a flexible parametric survival regression model with all four lifestyle factors in the entire population and all-cause mortality as the outcome. The original binary lifestyle variables for each participant were then multiplied by the standardised weighted β coefficients. The distribution of the weighted score is shown below, ranging from less healthy (0) to healthier (1).

**Figure S2.A**. Distribution of the weighted healthy lifestyle score for the entire population

The coefficients estimated in a random third of the population are consistent with those estimated in the whole population and are shown in the table below. Delta is the difference between the β coefficient for entire population and from the random third of the population.

| Healthy lifestyle factor in the model | **β coefficient**  **from entire population** | **β coefficient from random 1/3 of population** | **Delta** | **exp^Delta^** |
| --- | --- | --- | --- | --- |
| Regular physical activity | -0.2923622 | -0.2838981 | -0.0084641 | 0.9915716 |
| No current smoking | -0.9015466 | -0.9007862 | -0.0007604 | 0.9992399 |
| None/moderate alcohol consumption | -0.0798640 | -0.1285357 | 0.0486717 | 1.0498756 |
| Healthy diet | -0.1535554 | -0.1866419 | 0.0330865 | 1.0336399 |

In a sensitivity analysis, the above β coefficients obtained from the random one third of population (N=160,313) were standardised and applied to the remaining two thirds of the sample to the estimate the weighted score (N=320,627).

| Healthy lifestyle factor in the model | **β coefficient**  **from random 1/3 of population** | **Weighted**  **β coefficient** |
| --- | --- | --- |
| Regular physical activity (Yes vs No) | -0.2838981 | 0.189282827 |
| No current smoking (Yes vs No) | -0.9007862 | 0.600579427 |
| None/moderate alcohol consumption (Yes vs No) | -0.1285357 | 0.085698357 |
| Healthy diet (Yes vs No) | -0.1866419 | 0.12443939 |
| Total | -1.4998619 | 1 |

The distribution of the weighted score for the remaining 2/3 of the sample is shown below, ranging from less healthy (0) to healthier (1).

**Figure S2.B**. Distribution of the weighted healthy lifestyle score for 2/3 of the population
