# Supplementary material for: Healthy lifestyle and life expectancy in people with multimorbidity in the UK Biobank: A longitudinal cohort study
Source: PLoS Med. 2020 Sep 22;17(9):e1003332. doi: 10.1371/journal.pmed.1003332 (PMC7508366; doi:10.1371/journal.pmed.1003332)
Supplement: S3 Text — (DOCX) [file pmed.1003332.s003.docx]

# **S3 Text:** Missing lifestyle and covariate data

Missing lifestyle and covariate data were imputed using a single imputation approach. The single imputation method was chosen over the multiple imputation, because we were unable to process survival curves required for the life expectancy calculations due to the computational time using an extremely large database. We replaced the missing values using the observed data. This information was taken from participants with complete data where the mean value was used to impute continuous data and modal value was used to impute categorical data.

Due to the large number of participants missing a value for the leisure-time physical activity measure (n=11,517; 2.3%), we did not impute the missing value for these participants.

**Table S3.A** presents the number and percentage missing, the method, and the value that was imputed. The weighted healthy lifestyle score that includes the missing data is provided in **Table S3.B**.

**Table S3.A** Number and method of missing data

| Characteristic | **No. missing (%)** | **Imputation method** | **Value imputed** |
| --- | --- | --- | --- |
| Smoking status | 1,768 (0.4) | Mode | Never/ previous smoker |
| Healthy diet | 555 (0.1) | Mode | No |
| Alcohol consumption | 612 (0.1) | Mode | Never or <14 units/wk |
| Ethnicity | 1,684 (0.3) | Mode | White |
| Body mass index | 2,265 (0.5) | Mean | 27.40477 kg/m^2^ |
| Deprivation | 608 (0.1) | Mean | -1.324796 |
| Sedentary behaviour | 459 (0.1) | Mean | 5.079754 hours |

% calculated from the total number of participants.

Participants may have more than one missing covariates.

**Table S3.B** Weighted healthy lifestyle score including missing data (full sample)

| Healthy lifestyle factor in the model | **β coefficient** | **Weighted**  **β coefficient** |
| --- | --- | --- |
| Regular physical activity (Yes vs No) | -0.2985672 | 0.208374 |
| No current smoking (Yes vs No) | -0.9061541 | 0.632416 |
| None /moderate alcohol consumption (Yes vs No) | -0.0695284 | 0.048525 |
| Healthy diet (Yes vs No) | -0.1585964 | 0.110686 |
| Total | -1.4328461 | 1 |
