# Supplementary material for: Healthy lifestyle and life expectancy in people with multimorbidity in the UK Biobank: A longitudinal cohort study
Source: PLoS Med. 2020 Sep 22;17(9):e1003332. doi: 10.1371/journal.pmed.1003332 (PMC7508366; doi:10.1371/journal.pmed.1003332)
Supplement: S4 Text — (DOCX) [file pmed.1003332.s004.docx]

# **S4 Text:** Continuous weighted healthy lifestyle score

We re-analysed all data using a continuous score obtained from continuous variables. The following steps were undertaken:

1. The 3 continuous components of the score (physical activity in metabolic equivalents [METs]; units of alcohol per week; and daily portions of fruit/vegetables] were modelled as continuous variables instead of using above/below guidelines-defined thresholds.
2. Two survival models (with time to death as outcome) were then compared:
3. with 3 continuous linear variables + smoking (available as former/current/never);
4. with transformation of the 3 variables using restricted cubic spline with knots at the 10^th^, 30^th^, 50^th^, 70^th^, 90^th^ centile of their distributions + smoking

The models (a) and (b) were then compared with a (partial) likelihood ratio test, which indicated a “statistically significant” difference (p<0.05) between the two models; both the AIC and the BIC indicated that the model with continuous non-linear variables was “better” (i.e., lower values).

1. We then ran model (b) and estimated the linear predictor (log hazard ratio), which is the individual “lifestyle score”. For ease of interpretation, we rescaled the score from 0 to 1 (**Figure S4.A**). Coefficients are shown in **Table S4.A**.

This score was then used in all subsequent parametric Royston-Parmar survival analyses to estimate the residual life expectancy and years of life lost. We made predictions for 0.1 unit increase of score from 0 to 1 to plot the mean estimated residual life vs score and, given the large computational time, estimated uncertainties (i.e., 95% CI) at values of score of 0, 0.2, 0.4, 0.6, 0.8, and 1. To assess the robustness of the results, we also estimated the score in a random 1/3 of the sample and applied it to the remaining 2/3 (**Figure S4.A**; **Table S4.A**); and recalculated the score after imputing missing data.

**Figure S4.A.** Distribution of the continuous weighted healthy lifestyle score

**Table S4.A**: Coefficients from the lifestyle score

| Healthy lifestyle factor in the model | **β coefficient**  **from entire population** | **β coefficient from random 1/3 of population** | **β coefficient from entire population including entire population** |
| --- | --- | --- | --- |
| Regular physical activity (per 1-unit increase in METs) |  |  |  |
| Spline 1 | -0.0067616 | -0.0035988 | -0.0068832 |
| Spline 2 | -0.000012 | -4.45e-06 | -0.0000113 |
| Spline 3 | 0.0000222 | 0.0000151 | 0.0000214 |
| Spline 4 | -0.0000194 | -0.0000166 | -0.0000192 |
| Alcohol consumption (per 1-unit increase per week) |  |  |  |
| Spline 1 | -0.0061892 | -0.0030078 | -0.0068912 |
| Spline 2 | -0.0000347 | -0.0000225 | -0.0000352 |
| Spline 3 | 0.0000302 | 0.0000185 | 0.0000298 |
| Spline 4 | -8.69e-06 | -4.88e-06 | -7.83e-06 |
| Healthy diet (per 1-unit increase in daily portions of fruit/vegetables) |  |  |  |
| Spline 1 | -0.032161 | -0.04364 | -0.0325041 |
| Spline 2 | -0.0001804 | -0.0003725 | -0.0002158 |
| Spline 3 | 0.0001607 | 0.0004895 | 0.0002502 |
| Spline 4 | -0.000031 | -0.0002947 | -0.0001267 |
| No current smoking (Ref. Current smoking) | -0.8167038 | -0.8188641 | -0.8192026 |
