# Supplementary material for: Healthy lifestyle and life expectancy in people with multimorbidity in the UK Biobank: A longitudinal cohort study
Source: PLoS Med. 2020 Sep 22;17(9):e1003332. doi: 10.1371/journal.pmed.1003332 (PMC7508366; doi:10.1371/journal.pmed.1003332)
Supplement: S5 Text — (DOCX) [file pmed.1003332.s005.docx]

# **S5 Text:** Matching

Before matching, there were 480,940 participants (93,746 with multimorbidity; 387,194 without multimorbidity; **Table 1**); after matching, all participants with multimorbidity were matched to 74,013 participants without multimorbidity. The single nearest-neighbour without caliper matching procedure was performed in Stata 16.0 using the *psmatch2* command (version 4.0.12); the distribution of the propensity score is shown in **Figure S5.A**.

**Figure S5.A.** Distribution of the propensity
