# Supplementary material for: Healthy lifestyle and life expectancy in people with multimorbidity in the UK Biobank: A longitudinal cohort study
Source: PLoS Med. 2020 Sep 22;17(9):e1003332. doi: 10.1371/journal.pmed.1003332 (PMC7508366; doi:10.1371/journal.pmed.1003332)
Supplement: S1 Table — (DOCX) [file pmed.1003332.s006.docx]

# S1 Table: Previous studies investigating combined lifestyle factors and life expectancy

| **Reference** | **Study characteristics** | **Study population** | **Lifestyles factors** | **Method of calculating the combined healthy lifestyle score** | **Adjusted confounders when estimating the mortality risk** | **Key findings: Difference in years of life from the lowest to highest group (95% CI)** |
| --- | --- | --- | --- | --- | --- | --- |
| **Zaninotto et al. 2020 (1)** | N=17,351, England and United States  Average follow-up:  6 y  Starting age=50 y | General, also disability-free, chronic disease-free | 1. Smoking 2. Body mass index 3. Physical activity 4. Alcohol intake | Dichotomised  High-risk factor=1  Low-risk factor=0  Score was summed, ranged 0, 1, 2, and more. Higher scores indicating a unhealthy lifestyle | Stratified by sex, country | General  England  8.0 y men  7.6 y women  United States  8.1 y men  7.1 y women |
| **Nyberg et al. 2020 (2)** | N= 116 043, 12 European cohorts  Average follow-up: 12.5 y  Starting age=40 y | Free of major non-communicable disease at baseline | 1. Smoking 2. Body mass index 3. Physical activity 4. Alcohol intake | Aggregating responses for the individual lifestyle factors: optimal (2 points), intermediate (1 point), or poor (0 points)  Score ranging from 0 (lowest healthy score, highest risk) to 8 (highest healthy score, lowest risk) | Stratified by sex | 9.9 (6.7 to 13.1) y men  9.4 (5.4 to 13.3) y women |
| **Li et al. 2020 (3)** | N=123,219, United States  Average follow-up: 28 y  Starting age=50 y | Free from cancer, cardiovascular disease (CVD), type 2 diabetes | 1. Smoking 2. Body mass index 3. Physical activity 4. Alcohol intake 5. Diet | Dichotomised  Low-risk factor=1  High-risk factor=0  Score was summed, ranged 0 to 5, higher scores indicating a healthier lifestyle | Ethnicity, current multivitamin use, current aspirin use, family history of diabetes mellitus, myocardial infarction, or cancer, and menopausal status and hormone use (women only) | Free of cancer, CVD, type 2 diabetes:  7.6 (6.8 to 8.4) y men  10.6 (10.0, 11.3) y women  Free of cancer:  6.0 (5.4 to 6.7) y men  8.3 (7.8 to 8.9) y women  Free of CVD:  8.6 (7.9 to 9.4) y men  10.0 (9.3 to 10.6) y women  Free of type 2 diabetes:  10.3 (9.6 to 11.1) y men  12.3 (11.4 to 13.4) y women |
| **Reference** | **Study characteristics** | **Study population** | **Lifestyles factors** | **Method of calculating the combined healthy lifestyle score** | **Adjusted confounders when estimating the mortality risk** | **Key findings: Difference in years of life from the lowest to highest group (95% CI)** |
| **Xiong-Fei et al. 2019 (4)** | N=44,052, China  Average follow-up: 20.6 y  Starting age= 50 y | General | 1. Smoking 2. Body mass index 3. Physical activity 4. Alcohol intake 5. Diet | Dichotomised  Low-risk factor=1  High-risk factor=0  Score was summed, ranged 0 to 5, higher scores indicating a healthier lifestyle | Age and sex | 6.6 (4.9, 8.4) y men  8.1 (5.8, 10.5) y women |
| **Li et al. 2018 (5)** | N=123,219, United States  Average follow-up: 34 y  Starting age=50 y | General | 1. Smoking 2. Body mass index 3. Physical activity 4. Alcohol intake 5. Diet | Dichotomised  Low-risk factor=1  High-risk factor=0  Score was summed, ranged 0 to 5, higher scores indicating a healthier lifestyle | Ethnicity, current multivitamin use, current aspirin use, family history of diabetes mellitus, myocardial infarction, or cancer, and menopausal status and hormone use (women only) | 14.0 (11.8,16.2) y men  12.2 (10.1, 14.2) y women |
| **Manuel et al.**  **2016 (6)** | N= 77,399, Canada  Average follow-up: 7.6 y  Starting age: 20 y | General | 1. Smoking 2. Physical activity 3. Diet 4. Alcohol consumption | Mortality risks was estimated for each lifestyle and used to predict life expectancy in the lifetables  Grouped into two categories:  1) healthy profile (only included if had all four healthy lifestyles)  versus  2) unhealthy profile (only included if had all four unhealthy lifestyles) | Age, health behaviours, sociodemographic, and disease indicators | 16.8 y men  18.9 y women |

| **Reference** | **Study characteristics** | **Study population** | **Lifestyles factors** | **Method of calculating the combined healthy lifestyle score** | **Adjusted confounders when estimating the mortality risk** | **Key findings: Difference in years of life from the lowest to highest group (95% CI)** |
| --- | --- | --- | --- | --- | --- | --- |
| **O’Doherty et al.**  **2015 (7)** | N= 24,731, Denmark, Germany, Norway  Average follow-up: 4.5 y  Starting age: 50 y | General, also stratified with and without cardiovascular disease | 1. Smoking 2. Physical activity 3. Obesity 4. Alcohol consumption | Grouped into two lifestyle categories:  1) favourable (only included if had all four favourable lifestyles)  versus  2) unfavourable (only included if had all four unfavourable lifestyles) | Age, history of diabetes, hypertension and total/HDL cholesterol ratio | Denmark  9.2 (6.8, 11.6) y men  9.7 (7.4, 12.6) y women  Germany  15.1 (11.0, 20.4) y men,  15.7 (11.7, 19.8) y women  Norway  7.4 (6.3, 8.4) y men,  7.8 (6.8, 9.0) y women |
| **Kuanrong et al. 2014 (8)** | N=22,469, Germany  Average follow-up: 11 y  Starting age: 40 y | General | 1. Smoking 2. Body mass index 3. Physical activity 4. Alcohol intake   Diet | Calculated the combined effect:  1) favourable (only included if had all five favourable lifestyles)  versus  2) unfavourable (only included if had all five unfavourable lifestyles) | Education,  self-reported hypertension and hyperlipidaemia | 17.0 y men  13.9 y women |
| **Manuel et al. 2012 (9)** | N=78,597, Canada  Average follow-up: 568,997 person-years  Starting age: 20 y | General | 1. Smoking 2. Physical activity 3. Alcohol intake 4. Diet 5. Stress | Mortality risks was estimated for each lifestyle and used to predict life expectancy in the lifetables  Grouped into two categories:  1) healthy profile (only included if had all four healthy lifestyles)  versus  2) unhealthy profile (only included if had all four unhealthy lifestyles) | Age, ethnicity, education, rurality,  neighbourhood deprivation | 7.5 y |

| **Reference** | **Study characteristics** | **Study population** | **Lifestyles factors** | **Method of calculating the combined healthy lifestyle score** | **Adjusted confounders when estimating the mortality risk** | **Key findings: Difference in years of life from the lowest to highest group (95% CI)** |
| --- | --- | --- | --- | --- | --- | --- |
| **Rizzuto et al.**  **2012 (10)** | N= 1,810, Sweden  Average follow-up:  18 y  Starting age: 75 y | Adults over the age of 75 years, stratified by one or more chronic diseases and no chronic disease | 1. Smoking 2. Physical activity 3. Diet 4. Social network | Compared the median age at death for each group | Age, education, sex and number of chronic conditions if applicable | - 1. y whole population   6.3 y men  5.3 y women  3.0 y no chronic disease  4.7 y one or more chronic disease |
| **Lee et al.**  **2011 (11)** | N= 24,731, United States  Average follow-up: 14.5 y  Starting age: 20 y | General,  men | 1. Smoking 2. Moderate or high fitness 3. Normal waist | Dichotomised  Low-risk factor=1  High-risk factor=0  Score was summed, ranged 0 to 3, higher scores indicating a healthier lifestyle | Age, examination year, alcohol intake, and family history of cancer | 12.0 (8.6,14.6) y men |
| **Tamakoshi et al. 2010 (12)** | N=62,106, Japan  Average follow-up:14.5 y  Starting age: 40 y | General | 1. Smoking 2. Body mass index 3. Walking duration 4. Sleep duration 5. Alcohol consumption 6. Diet: consumption of green leafy vegetables | Dichotomised  Low-risk factor=1  High-risk factor=0  Score was summed, ranged 0 to 6, higher scores indicating a healthier lifestyle | Age-adjusted | 10.3 y men  8.3 y women |

| **Reference** | **Study characteristics** | **Study population** | **Lifestyles factors** | **Method of calculating the combined healthy lifestyle score** | **Adjusted confounders when estimating the mortality risk** | **Key findings: Difference in years of life from the lowest to highest group (95% CI)** |
| --- | --- | --- | --- | --- | --- | --- |
| **Khaw et al.**  **2004 (13)** | N= 25,639, Norfolk, United Kingdom  Average follow-up: 11 y  Starting age: 45 y | General | 1. Smoking 2. Physical activity 3. Alcohol consumption 4. Diet: Fruit and vegetable intake (based on plasma vitamin C level >50 mmol/l) | Dichotomised  Low-risk factor=1  High-risk factor=0  Score was summed, ranged 0 to 4, higher scores indicating a healthier lifestyle | Age, sex, body mass index, and social class | 14.0 y |

Y=years; CI=confidence interval.

Google Scholar and PubMed search was carried out with the terms “lifestyle factors" AND “life expectancy” on 9^th^ October 2019 and updated on 6^th^ June 2020. In the table we reported the studies we deemed most relevant. Studies are presented in reverse chronological order.

**References**

1. Zaninotto P, Head J, Steptoe A. Behavioural risk factors and healthy life expectancy: evidence from two longitudinal studies of ageing in England and the US. Scientific Reports. 2020;10(1):1-9.

2. Nyberg ST; Singh-Manoux A; Pentti J MI, Sabia S, Alfredsson L, et al. Association of Healthy Lifestyle With Years Lived Without Major Chronic Diseases. JAMA Internal Medicine. 2020;180(5):760-8.

3. Li Y, Schoufour J, Wang DD, Dhana K, Pan A, Liu X, et al. Healthy lifestyle and life expectancy free of cancer, cardiovascular disease, and type 2 diabetes: prospective cohort study. BMJ. 2020;368:l6669.

4. Pan XF, Li Y, Franco OH, Yuan JM, Pan A, Koh WP. Impact of combined lifestyle factors on all-cause and cause-specific mortality and life expectancy in Chinese: the Singapore Chinese Health Study. J Gerontol A Biol Sci Med Sci. 2019.

5. Li Y, Pan A, Wang DD, Liu X, Dhana K, Franco OH, et al. Impact of Healthy Lifestyle Factors on Life Expectancies in the US Population. Circulation. 2018;138(4):345-55.

6. Manuel DG, Perez R, Sanmartin C, Taljaard M, Hennessy D, Wilson K, et al. Measuring Burden of Unhealthy Behaviours Using a Multivariable Predictive Approach: Life Expectancy Lost in Canada Attributable to Smoking, Alcohol, Physical Inactivity, and Diet. PLoS Medicine. 2016;13(8):e1002082.

7. O’Doherty MG, Cairns K, O’Neill V, Lamrock F, Jørgensen T, Brenner H, et al. Effect of major lifestyle risk factors, independent and jointly, on life expectancy with and without cardiovascular disease: results from the Consortium on Health and Ageing Network of Cohorts in Europe and the United States (CHANCES). European Journal of Epidemiology. 2016;31(5):455-68.

8. Li K, Hüsing A, Kaaks R. Lifestyle risk factors and residual life expectancy at age 40: a German cohort study. BMC Medicine. 2014;12(1):59.

9. Manuel DG, Perez R, Bennett C, Rosella L, Taljaard M, Roberts M, et al. Seven more years: the impact of smoking, alcohol, diet, physical activity and stress on health and life expectancy in Ontario: Toronto. Institute for Clinical Evaluative Sciences and Public Health Ontario. 2012.

10. Rizzuto D, Orsini N, Qiu C, Wang H-X, Fratiglioni L. Lifestyle, social factors, and survival after age 75: population based study. BMJ : British Medical Journal. 2012;345:e5568.

11. Lee C-D, Sui X, Hooker SP, Hébert JR, Blair SN. Combined impact of lifestyle factors on cancer mortality in men. Ann Epidemiol. 2011;21(10):749-54.

12. Tamakoshi A, Kawado M, Ozasa K, Tamakoshi K, Lin Y, Yagyu K, et al. Impact of smoking and other lifestyle factors on life expectancy among japanese: findings from the Japan Collaborative Cohort (JACC) Study. Journal of epidemiology. 2010;20(5):370-6.

13. Khaw K-T, Wareham N, Bingham S, Welch A, Luben R, Day N. Combined Impact of Health Behaviours and Mortality in Men and Women: The EPIC-Norfolk Prospective Population Study. PLoS Medicine. 2008;5(1):e12.
