# Supplementary material for: Healthy lifestyle and life expectancy in people with multimorbidity in the UK Biobank: A longitudinal cohort study
Source: PLoS Med. 2020 Sep 22;17(9):e1003332. doi: 10.1371/journal.pmed.1003332 (PMC7508366; doi:10.1371/journal.pmed.1003332)
Supplement: S2 Table — (DOCX) [file pmed.1003332.s007.docx]

# **S2 Table**: Summary of main and sensitivity analyses

| **Analysis** | **Multimorbidity** | **Sample** | **Exposure** | **Table/Figure** |
| --- | --- | --- | --- | --- |
| Main analysis | 2+ conditions | Complete-case | Main weighted score ^a^ | Figure 1 & 2, Table 2 |
|  |  | Complete-case | Individual risk factor | Table 3 |
| Sensitivity analysis - 1 | 2+ conditions | Complete-case | Main weighted score from 1/3 data ^b^ | S5 Table |
|  |  | Imputation ^c^ | Main weighted score ^a^ | S6 Table |
|  |  | Imputation ^c^ | Individual risk factor | S7 Table |
| Sensitivity analysis - 2 | 2+ conditions | Complete-case | Continuous weighted score ^d^ | S2 Figure, S8 Table |
|  |  | Complete-case | Continuous weighted score from 1/3 data ^d^ | S3 Figure, S9 Table |
|  |  | Imputation ^c^ | Continuous weighted score ^d^ | S4 Figure, S10 Table |
| Sensitivity analysis - 3 | Cardiometabolic* | Complete-case | Main weighted score ^a^ | S11 Table |
|  |  | Complete-case | Individual risk factor | S12 Table |
| Sensitivity analysis - 4 | 2+ conditions | Complete-case | Score 0-4 (sum risk factors) | S13 Table |
|  | Cardiometabolic* | Complete-case | Score 0-4 (sum risk factors) | S14 Table |
| Sensitivity analysis - 5 | 2+ conditions | Complete-case  matched cohort ^e^ | Main weighted score ^a^ | S5 Figure, S15 Table |

* Diabetes + CVD (stroke, myocardial infarction, heart failure, angina or peripheral vascular disease)

^a^ Coefficients quantified using categorical risk factors and subjects classified as very unhealthy (score 0-0.25; reference group), unhealthy (≥0.25-0.50), healthy (≥0.50-0.75), and very healthy (≥0.75-1). Details are reported in **S2 Methods**.

^b^ Main weighted score derived in random 1/3 data and applied to remaining 2/3. Details are reported in **S2 Methods**.

^c^ Details are reported in **S3 Methods**.

^d^ Coefficients quantified using continuous risk factors and categorical smoking. Details are reported in **S4 Methods**.

^e^ Details are reported in **S5 Methods**.
