# Supplementary material for: Healthy lifestyle and life expectancy in people with multimorbidity in the UK Biobank: A longitudinal cohort study
Source: PLoS Med. 2020 Sep 22;17(9):e1003332. doi: 10.1371/journal.pmed.1003332 (PMC7508366; doi:10.1371/journal.pmed.1003332)
Supplement: S3 Table — (DOCX) [file pmed.1003332.s008.docx]

# **S3 Table**: Most to least prevalent chronic conditions, by sex

| Men | | | |  | Women | | | |
| --- | --- | --- | --- | --- | --- | --- | --- | --- |
|  | Chronic condition | No. | % |  |  | Chronic condition | No. | % |
| 1 | Hypertension | 64,774 | 29.6 |  | **1** | Hypertension | 59386 | 22.7 |
| 2 | Asthma | 23,369 | 10.7 |  | **2** | Asthma | 32172 | 12.3 |
| 3 | Cancer | 13,754 | 6.3 |  | **3** | Cancer | 25562 | 9.8 |
| 4 | Diabetes | 12,610 | 5.8 |  | **4** | Depression | 17660 | 6.7 |
| 5 | Angina | 10,033 | 4.6 |  | **5** | Migraine | 10897 | 4.2 |
| 6 | Depression | 9,101 | 4.2 |  | **6** | Irritable bowel syndrome | 8282 | 3.2 |
| 7 | Myocardial infarction | 8,488 | 3.9 |  | **7** | Diabetes | 7418 | 2.8 |
| 8 | Eczema or dermatitis | 5,448 | 2.5 |  | **8** | Osteoporosis | 6732 | 2.6 |
| 9 | Stroke | 3,540 | 1.6 |  | **9** | Eczema or dermatitis | 6669 | 2.5 |
| 10 | Migraine | 3,036 | 1.4 |  | **10** | Angina | 4845 | 1.8 |
| 11 | Irritable bowel syndrome | 2,797 | 1.3 |  | **11** | Anxiety or panic attacks | 4132 | 1.6 |
| 12 | Glaucoma | 2,642 | 1.2 |  | **12** | Rheumatoid arthritis | 3686 | 1.4 |
| 13 | Atrial fibrillation | 2,369 | 1.1 |  | **13** | Stroke | 2445 | 0.9 |
| 14 | Anxiety or panic attacks | 2,291 | 1.0 |  | **14** | Glaucoma | 2405 | 0.9 |
| 15 | Vestibular disorder | 2,035 | 0.9 |  | **15** | Myocardial infarction | 2081 | 0.8 |
| 16 | Epilepsy | 1,829 | 0.8 |  | **16** | Vestibular disorder | 2021 | 0.8 |
| 17 | Rheumatoid arthritis | 1,580 | 0.7 |  | **17** | Epilepsy | 1904 | 0.7 |
| 18 | Chronic sinusitis | 1,202 | 0.5 |  | **18** | Chronic sinusitis | 1793 | 0.7 |
| 19 | Tuberculosis | 1,069 | 0.5 |  | **19** | Tuberculosis | 1307 | 0.5 |
| 20 | Meningitis | 928 | 0.4 |  | **20** | Atrial fibrillation | 1092 | 0.4 |
| 21 | Osteoporosis | 849 | 0.4 |  | **21** | Meningitis | 981 | 0.4 |
| 22 | Prostate problem | 802 | 0.4 |  | **22** | Multiple sclerosis | 1080 | 0.4 |
| 23 | COPD | 729 | 0.3 |  | **23** | Thyroid problem | 1064 | 0.4 |
| 24 | Parkinson’s disease | 497 | 0.2 |  | **24** | COPD | 783 | 0.3 |
| 25 | Multiple sclerosis | 378 | 0.2 |  | **25** | Bronchiectasis | 714 | 0.3 |
| 26 | Schizophrenia | 368 | 0.2 |  | **26** | Peripheral vascular disease | 545 | 0.2 |
| 27 | Bronchiectasis | 367 | 0.2 |  | **27** | Anaemia | 631 | 0.2 |
| 28 | Peripheral vascular disease | 334 | 0.2 |  | **28** | Parkinson’s disease | 290 | 0.1 |
| 29 | Hepatitis | 269 | 0.1 |  | **29** | Schizophrenia | 180 | 0.1 |
| 30 | Anaemia | 214 | 0.1 |  | **30** | Hepatitis | 248 | 0.1 |
| 31 | Thyroid problem | 192 | 0.1 |  | **31** | Cirrhosis | 163 | 0.1 |
| 32 | Heart failure | 173 | 0.1 |  | **32** | Chronic kidney disease | 99 | 0.0 |
| 33 | Cirrhosis | 151 | 0.1 |  | **33** | Heart failure | 95 | 0.0 |
| 34 | Chronic kidney disease | 113 | 0.1 |  | **34** | Dementia | 50 | 0.0 |
| 35 | Inflammatory bowel disease | 69 | 0.0 |  | **35** | Prostate problem | 0 | 0.0 |
| 36 | Dementia | 63 | 0.0 |  | **36** | Chronic kidney disease | 99 | 0.0 |
