# Supplementary material for: Healthy lifestyle and life expectancy in people with multimorbidity in the UK Biobank: A longitudinal cohort study
Source: PLoS Med. 2020 Sep 22;17(9):e1003332. doi: 10.1371/journal.pmed.1003332 (PMC7508366; doi:10.1371/journal.pmed.1003332)
Supplement: S4 Table — (DOCX) [file pmed.1003332.s009.docx]

# **S4 Table:** Number of participants by total number of chronic conditions

| Total number of conditions | Frequency | % | Cumulative % |
| --- | --- | --- | --- |
| 0 | 224,532 | 46.69 | 46.69 |
| 1 | 162,662 | 33.82 | 80.51 |
| 2 | 65,903 | 13.70 | 94.21 |
| 3 | 20,386 | 4.24 | 98.45 |
| 4 | 5,532 | 1.15 | 99.60 |
| 5 | 1,470 | 0.31 | 99.91 |
| 6 | 334 | 0.07 | 99.97 |
| 7 | 102 | 0.02 | 100 |
| 8 | 17 | 0 | 100 |
| 9 | 1 | 0 | 100 |
| 10 | 1 | 0 | 100 |
