# Supplementary material for: Healthy lifestyle and life expectancy in people with multimorbidity in the UK Biobank: A longitudinal cohort study
Source: PLoS Med. 2020 Sep 22;17(9):e1003332. doi: 10.1371/journal.pmed.1003332 (PMC7508366; doi:10.1371/journal.pmed.1003332)
Supplement: S5 Table — (DOCX) [file pmed.1003332.s010.docx]

# S5 Table: Survival using the weighted score obtained from a random one third of the population

| Healthy lifestyle category | With multimorbidity | | Without multimorbidity | |
| --- | --- | --- | --- | --- |
|  | **Men**  (n=28,879) | **Women**  (n=33,587) | **Men**  (n=116,897) | **Women**  (n=141,264) |
| No. deaths / No. participants | | | | |
| Very unhealthy | 295 / 2,747 | 151 / 2463 | 504 / 10,665 | 220 / 8,674 |
| Unhealthy | 66 / 737 | 30 / 728 | 111 / 3,679 | 78 / 3,540 |
| Healthy | 639 / 10,020 | 386 / 11,717 | 763 / 34,139 | 556 / 44,792 |
| Very healthy | 742 / 15,375 | 526 / 18,679 | 1,291 / 68,414 | 1,071 / 84,258 |
|  |  |  |  |  |
| HR (95% CI) | | | | |
| Very unhealthy | 1 (Reference) | 1 (Reference) | 1 (Reference) | 1 (Reference) |
| Unhealthy | 0.86 (0.66, 1.13) | 0.70 (0.47, 1.03) | 0.63 (0.51, 0.77) | 0.87 (0.67, 1.13) |
| Healthy | 0.55 (0.48, 0.63) | 0.50 (0.41, 0.61) | 0.43 (0.38, 0.48) | 0.44 (0.38, 0.52) |
| Very healthy | 0.42 (0.36, 0.48) | 0.41 (0.34, 0.50) | 0.35 (0.32, 0.39) | 0.42 (0.36, 0.49) |
|  |  |  |  |  |
| Years of life gained [95% CI], 45 y | | | | |
| Very unhealthy | Reference | Reference | Reference | Reference |
| Unhealthy | 1.11 [-0.95, 3.17)] | 3.27 [-0.22, 6.76] | 3.30 [1.69, 4.91] | 1.04 [-0.95, 3.04] |
| Healthy | 4.47 [3.12, 6.02] | 6.02 [4.14, 7.89] | 6.03 [4.77, 7.29] | 5.88 [4.49, 7.28] |
| Very healthy | 6.64 [4.97, 8.30] | 7.40 [5.54, 9.26] | 7.44 [6.01, 8.78] | 6.17 [4.81, 7.52] |
|  |  |  |  |  |
| Years of life gained [95% CI], 65 y | | | | |
| Very unhealthy | Reference | Reference | Reference | Reference |
| Unhealthy | 0.87 [-0.76, 2.50] | 2.75 [-0.20, 5.70] | 2.83 [1.41, 4.25] | 0.92 [-0.85, 2.70] |
| Healthy | 3.71 [2.48, 4.93] | 5.09 [3.49, 6.69] | 5.28 [4.12, 6.43] | 5.30 [4.03, 6.57] |
| Very healthy | 5.48 [4.04, 6.92] | 6.28 [4.68, 7.88] | 6.56 [5.32, 7.80] | 5.56 [4.32, 6.79] |

Y=years; p=participants; HR=hazard ratio; CI=confidence intervals. Sample size of the remaining 2/3 of the population: N=320,627.

Models adjusted for ethnicity (white, non-white), working status (working, retired, other), deprivation (continuous), body mass index (continuous), sedentary time (continuous).
