# Supplementary material for: Healthy lifestyle and life expectancy in people with multimorbidity in the UK Biobank: A longitudinal cohort study
Source: PLoS Med. 2020 Sep 22;17(9):e1003332. doi: 10.1371/journal.pmed.1003332 (PMC7508366; doi:10.1371/journal.pmed.1003332)
Supplement: S6 Table — (DOCX) [file pmed.1003332.s011.docx]

# S6 Table: Survival using the weighted score following imputation of missing data

| Healthy lifestyle category | With multimorbidity | | Without multimorbidity | |
| --- | --- | --- | --- | --- |
|  | **Men**  (n=44,430) | **Women**  (n=51,161) | **Men**  (n=178,351) | **Women**  (n=214,533) |
| No. deaths / No. participants | | | | |
| Very unhealthy | 438 / 4,208 | 236 / 3,798 | 773 / 16,351 | 340 / 13,020 |
| Unhealthy | 99 / 1,187 | 46 / 1,164 | 183 / 5,665 | 119 / 5,403 |
| Healthy | 981 / 15,408 | 562 / 17,930 | 1,228 / 52,163 | 848 / 68,206 |
| Very healthy | 1,192 / 23,627 | 762 / 28,269 | 1,921 / 104,172 | 1,583 / 127,904 |
|  |  |  |  |  |
| HR (95% CI) | | | | |
| Very unhealthy | 1 (Reference) | 1 (Reference) | 1 (Reference) | 1 (Reference) |
| Unhealthy | 0.81 (0.65, 1.00) | 0.67 90.49, 0.92) | 0.67 (0.57, 0.79) | 0.84 (0.68, 1.04) |
| Healthy | 0.57 (0.51, 0.64) | 0.47 (0.40, 0.55) | 0.46 (0.42, 0.51) | 0.43 (0.38, 0.49) |
| Very healthy | 0.45 (0.40, 0.51) | 0.39 (0.34, 0.46) | 0.35 (0.32, 0.38) | 0.40 (0.36, 0.45) |
|  |  |  |  |  |
| Years of life gained [95% CI], 45 y | | | | |
| Very unhealthy | Reference | Reference | Reference | Reference |
| Unhealthy | 1.72 [-0.06, 3.50] | 3.49 [0.72, 6.27] | 2.88 [1.61, 4.15] | 1.33 [-0.30, 2.97] |
| Healthy | 4.45 [3.26, 5.63] | 6.41 [4.86, 7.96] | 5.62 [4.64, 6.61] | 6.07 [4.95, 7.19] |
| Very healthy | 6.34 [5.02, 7.67] | 7.70 [6.15, 9.24] | 7.61 [6.53, 8.68] | 6.53 [5.44, 7.62] |
|  |  |  |  |  |
| Years of life gained [95% CI], 65 y | | | | |
| Very unhealthy | Reference | Reference | Reference | Reference |
| Unhealthy | 1.37 [-0.07, 2.81] | 2.95 [0.58, 5.32] | 2.48 [1.36, 3.59] | 1.18 [-0.27, 2.63] |
| Healthy | 3.63 [2.63, 4.64] | 5.46 [4.13, 6.80] | 4.91 [4.02, 5.82] | 5.46 [4.44, 6.48] |
| Very healthy | 5.26 [4.11, 6.40] | 6.58 [5.24, 7.92] | 6.72 [5.73, 7.71] | 5.88 [4.88, 6.88] |

Y=years; p=participants; HR=hazard ratio; CI=confidence intervals. Sample size: N=488,475.

Models adjusted for ethnicity (white, non-white), working status (working, retired, other), deprivation (continuous), body mass index (continuous), sedentary time (continuous).
