# Supplementary material for: Healthy lifestyle and life expectancy in people with multimorbidity in the UK Biobank: A longitudinal cohort study
Source: PLoS Med. 2020 Sep 22;17(9):e1003332. doi: 10.1371/journal.pmed.1003332 (PMC7508366; doi:10.1371/journal.pmed.1003332)
Supplement: S7 Table — (DOCX) [file pmed.1003332.s012.docx]

# S7 Table: Survival using individual lifestyle factor following imputation of missing data

| Healthy lifestyle factor | With multimorbidity | | Without multimorbidity | |
| --- | --- | --- | --- | --- |
|  | **Men**  (n=44,430) | **Women**  (n= 51,161) | **Men**  (n=178,351) | **Women**  (n=214,533) |
| Regular physical activity | | | | |
| No - No. of deaths/participants | 1,629 / 22,364 | 1,100 / 30,759 | 1,984 / 72,453 | 1,605 / 108,834 |
| Yes - No. of deaths/participants | 1,081 / 22,066 | 506 / 20,402 | 2,121 / 105,898 | 1,285 / 105,699 |
| HR (95% CI), Yes vs No (reference) | 0.72 (0.67, 0.78) | 0.77 (0.69, 0.86) | 0.77 (0.72, 0.82) | 0.87 (0.81, 0.94) |
| Years of life gained [95% CI], 45 y | 2.59 [1.85, 3.33] | 1.89 [1.10, 2.68] | 1.86 [1.36, 2.35] | 0.87 [0.39, 1.35] |
| Years of life gained [95% CI], 65 y | 2.20 [1.55, 2.84] | 1.63 [0.94, 2.31] | 1.67 [1.22, 2.13] | 0.79 [0.35, 1.23] |
|  |  |  |  |  |
| Smoking | | | | |
| Smoker - No. of deaths/participants | 537 / 5,395 | 282 / 4,962 | 956 / 22,016 | 459 / 18,423 |
| No current smoking - No. of deaths/participants | 2,173 / 39,035 | 1,324 / 46,199 | 3,149 / 156,335 | 2,431 / 196,110 |
| HR (95% CI), No vs Yes (reference) | 0.54 (0.49, 0.60) | 0.48 (0.41, 0.54) | 0.45 (0.41, 0.48) | 0.44 (0.40, 0.49) |
| Years of life gained [95% CI], 45 y | 4.86 [3.77, 5.95] | 6.00 [4.69, 7.31] | 5.87 [4.98, 6.75] | 5.82 [4.89, 6.76] |
| Years of life gained [95% CI], 65 y | 4.01 [3.08, 4.95] | 5.13 [4.00, 6.26] | 5.19 [4.38, 6.00] | 5.25 [4.39, 6.10] |
|  |  |  |  |  |
| Healthy diet | | | | |
| No - No. of deaths/participants | 1,851 / 29,539 | 945 / 28,611 | 2,954 / 123,447 | 1,608 / 121,485 |
| Yes - No. of deaths/participants | 859 / 14,891 | 661 / 22,550 | 1,151 / 54,904 | 1,282 / 93,048 |
| HR (95% CI), Yes vs No (reference) | 0.92 (0.85, 1.00) | 0.90 (0.81, 0.99) | 0.88 (0.82, 0.94) | 0.97 (0.90, 1.05) |
| Years of life gained [95% CI], 45 y | 0.63 [-0.03, 1.29] | 0.77 [0.04, 1.50] | 0.94 [0.44, 1.43] | 0.19 [-0.29, 0.66] |
| Years of life gained [95% CI], 65 y | 0.53 [-0.02, 1.09] | 0.66 [0.03, 1.30] | 0.84 [0.39, 1.29] | 0.17 [-0.26, 0.60] |
|  |  |  |  |  |
| Alcohol consumption | | | | |
| Excess - No. of deaths/participants | 1,183 / 20,927 | 288 / 10,951 | 2,194 / 92,674 | 710 / 57,934 |
| None/moderate - No. of deaths/participants | 1,527 / 23,503 | 1,318 / 40,210 | 1,911 / 85,677 | 2,180 / 156,599 |
| HR (95% CI), None/moderate vs Excess (reference) | 1.10 (1.02, 1.18) | 1.15 (1.01, 1.31) | 0.96 (0.90, 1.02) | 1.04 (0.95, 1.13) |
| Years of life gained [95% CI], 45 y | -0.73 [-1.35, -1.11] | -0.99 (-1.90, -0.08) | 0.28 [-0.16, 0.72] | -0.22 [-0.76, 0.32] |
| Years of life gained [95% CI], 65 y | -0.62 [-1.14, -0.09] | -0.85 [-1.64, -0.07] | 0.25 [-0.14, 0.65] | -0.20 [-0.69, 0.29] |

Y=years; p=participants; HR=hazard ratio; CI=confidence intervals; ref=reference. Sample size: N=488,475.

Regular physical activity: ≥500 MET-minutes/week; Healthy diet: at least five portions of fruit and vegetables every day; None/moderate alcohol consumption: 0 to 14 units of alcohol a week.

Models adjusted for ethnicity (white, non-white), working status (working, retired, other), deprivation (continuous), body mass index (continuous), sedentary time (continuous) and all other healthy lifestyle factors. The reference for years of life gained is the same used for hazard ratio.
