# Supplementary material for: Healthy lifestyle and life expectancy in people with multimorbidity in the UK Biobank: A longitudinal cohort study
Source: PLoS Med. 2020 Sep 22;17(9):e1003332. doi: 10.1371/journal.pmed.1003332 (PMC7508366; doi:10.1371/journal.pmed.1003332)
Supplement: S8 Table — CI, confidence interval (DOCX) [file pmed.1003332.s013.docx]

# S8 Table: Survival using the continuous weighted lifestyle score (confidence intervals)

| Healthy lifestyle continuous weighted score | With multimorbidity | | Without multimorbidity | |
| --- | --- | --- | --- | --- |
|  | **Men**  (n=43,448) | **Women**  (n=50,298) | **Men**  (n=175,380) | **Women**  (n=211,814) |
| HR (95% CI) ^a^ | | | | |
| Continuous weighted score | 1.04 (1.04, 1.05) | 1.05 (1.04, 1.06) | 1.05 (1.05, 1.06) | 1.05 (1.04, 1.06) |
|  |  |  |  |  |
| Score | **Estimated residual life expectancy [95% CI], 45 y** | | | |
| 0.0 | 44.53 [42.89, 46.17] | 49.66 [48.32, 51.00] | 47.42 [46.23, 48.61] | 50.41 [49.47, 51.36] |
| 0.2 | 38.04 [36.72, 39.36] | 43.55 [41.79, 45.32] | 40.70 [39.57, 41.83] | 45.20 [43.91, 46.48] |
| 0.4 | 31.29 [30.09, 32.50] | 35.02 [32.95, 37.08] | 33.33 [32.41, 34.25] | 37.85 [36.31, 39.38] |
| 0.6 | 24.68 [23.01, 26.36] | 26.36 [23.76, 28.96] | 26.20 [25.03, 27.38] | 30.22 [28.30, 32.14] |
| 0.8 | 18.15 [15.82, 20.49] | 18.37 [15.00, 21.75] | 19.16 [17.50, 20.82] | 22.92 [20.36, 25.48] |
| 1.0 | 12.06 [9.36, 14.76] | 11.33 [7.62, 15.04] | 12.50 [10.54, 14.45] | 15.95 [12.83, 19.08] |
|  |  |  |  |  |
| Score | **Estimated residual life expectancy [95% CI], 65 y** | | | |
| 0.0 | 25.35 [23.76, 26.95] | 30.23 [28.94, 31.51] | 27.81 [26.63, 28.98] | 30.73 [29.81, 31.65] |
| 0.2 | 19.62 [18.31, 20.93] | 24.83 [23.06, 26.61] | 21.59 [20.44, 22.73] | 25.93 [24.63, 27.23] |
| 0.4 | 14.20 [13.17, 15.23] | 17.61 [15.69, 19.53] | 15.21 [14.36, 16.06] | 19.37 [17.91, 20.84] |
| 0.6 | 9.76 [8.71, 10.80] | 11.16 [9.31, 13.00] | 9.95 [9.13, 10.76] | 13.20 [11.69, 14.71] |
| 0.8 | 6.33 [5.25, 7.41] | 6.51 [4.79, 8.22] | 5.96 [5.16, 6.77] | 8.32 [6.79, 9.85] |
| 1.0 | 3.83 [2.82, 4.83] | 3.48 [2.10, 4.86] | 3.22 [2.52, 3.91] | 4.80 [3.43, 6.17] |

Y=years; p=participants; HR=hazard ratio; CI=confidence intervals.

Models adjusted for ethnicity (white, non-white), working status (working, retired, other), deprivation (continuous), body mass index (continuous), sedentary time (continuous).

^a^ To make the HR interpretable, the score was rescaled to 0-100 (i.e., using the scale 0-1, the HR per 1-unit change would correspond to the comparison of the extremes).

The continuous score should be interpreted alongside the coefficients reported in **Table S4.A**.
