# Supplementary material for: Healthy lifestyle and life expectancy in people with multimorbidity in the UK Biobank: A longitudinal cohort study
Source: PLoS Med. 2020 Sep 22;17(9):e1003332. doi: 10.1371/journal.pmed.1003332 (PMC7508366; doi:10.1371/journal.pmed.1003332)
Supplement: S9 Table — CI, confidence interval (DOCX) [file pmed.1003332.s014.docx]

# S9 Table: Survival using the continuous weighted lifestyle score obtained from a random one third of the population (confidence intervals)

| Healthy lifestyle continuous weighted score | With multimorbidity | | Without multimorbidity | |
| --- | --- | --- | --- | --- |
|  | **Men**  (n=160,313) | **Women**  (n=33,587) | **Men**  (n=116,897) | **Women**  (n=141,264) |
| HR (95% CI) ^a^ | | | | |
| Continuous weighted score | 1.06 (1.05, 1.06) | 1.06 (1.05, 1.07) | 1.06 (1.06, 1.07) | 1.06 (1.05, 1.07) |
|  |  |  |  |  |
| Score | **Estimated residual life expectancy [95% CI], 45 y** | | | |
| 0.0 | 43.40 [41.41, 45.39] | 49.32 [47.74, 50.91] | 46.35 [44.83, 47.87] | 49.41 [48.10, 50.73] |
| 0.2 | 35.39 [34.02, 36.76] | 41.33 [39.09, 43.58] | 37.87 [36.66, 39.07] | 42.36 [40.68, 44.05] |
| 0.4 | 27.36 [25.72, 29.00] | 30.48 [27.48, 33.48] | 29.20 [28.03, 30.27] | 33.56 [31.49, 35.63] |
| 0.6 | 19.31 [16.71, 21.91] | 20.37 [16.35, 24.38] | 20.68 [18.87, 22.49] | 25.00 [22.01, 27.98] |
| 0.8 | 11.77 [8.63, 14.92] | 11.71 [7.07, 16.35] | 12.43 [10.13, 14.74] | 16.71 [12.75, 20.68] |
| 1.0 | 6.22 [3.58, 8.87] | 5.53 [1.86, 9.19] | 6.18 [4.26, 8.10] | 9.63 [5.77, 13.48] |
|  |  |  |  |  |
| Score | **Estimated residual life expectancy [95% CI], 45 y** | | | |
| 0.0 | 24.23 [22.29, 26.17] | 30.01 [28.49, 31.53] | 26.77 [25.27, 28.28] | 29.78 [28.49, 31.07] |
| 0.2 | 17.27 [15.94, 18.60] | 23.10 [20.86, 25.34] | 19.00 [17.79, 20.21] | 23.31 [21.62, 25.00] |
| 0.4 | 11.33 [10.17, 12.49] | 14.29 [11.81, 16.77] | 11.96 [11.02, 12.90] | 15.73 [13.94, 17.52] |
| 0.6 | 6.82 [5.57, 8.07] | 7.63 [5.36, 9.90] | 6.70 [5.76, 7.64] | 9.57 [7.66, 11.49] |
| 0.8 | 3.67 [2.50, 4.84] | 3.66 [1.91, 5.41] | 3.23 [2.43, 4.04] | 5.18 [3.40, 6.96] |
| 1.0 | 1.73 [0.88, 2.58] | 1.62 [0.54, 2.71] | 1.34 [0.84, 1.84] | 2.43 [1.11, 3.76] |

Y=years; p=participants; HR=hazard ratio; CI=confidence intervals. Sample size of the remaining 2/3 of the population: N=320,627.

Models adjusted for ethnicity (white, non-white), working status (working, retired, other), deprivation (continuous), body mass index (continuous), sedentary time (continuous).

^a^ To make the HR interpretable, the score was rescaled to 0-100 (i.e., using the scale 0-1, the HR per 1-unit change would correspond to the comparison of the extremes).

The continuous score should be interpreted alongside the coefficients reported in **Table S4.A**.
