# Supplementary material for: Healthy lifestyle and life expectancy in people with multimorbidity in the UK Biobank: A longitudinal cohort study
Source: PLoS Med. 2020 Sep 22;17(9):e1003332. doi: 10.1371/journal.pmed.1003332 (PMC7508366; doi:10.1371/journal.pmed.1003332)
Supplement: S10 Table — CI, confidence interval (DOCX) [file pmed.1003332.s015.docx]

# S10 Table: Survival using the continuous weighted lifestyle score following imputing missing data (confidence intervals)

| Healthy lifestyle continuous weighted score | With multimorbidity | | Without multimorbidity | |
| --- | --- | --- | --- | --- |
|  | **Men**  (n=44,430) | **Women**  (n=51,161) | **Men**  (n=178,351) | **Women**  (n=214,533) |
| HR (95% CI) ^a^ | | | | |
| Continuous weighted score | 1.04 (1.04, 1.05) | 1.05 (1.04, 1.06) | 1.05 (1.05, 1.06) | 1.05 (1.04, 1.06) |
|  |  |  |  |  |
| Score | **Estimated residual life expectancy [95% CI], 45 y** | | | |
| 0.0 | 44.22 [42.61, 45.83] | 49.55 [48.23, 50.87] | 47.13 [45.95, 48.31] | 50.27 [49.32, 51.21] |
| 0.2 | 37.81 [36.53, 39.08] | 43.41 [41.68, 45.15] | 40.43 [39.33, 41.53] | 45.02 [43.74, 46.30] |
| 0.4 | 31.19 [30.02, 32.36] | 34.91 [32.88, 36.93] | 33.17 [32.28, 34.07] | 37.72 [36.21, 39.23] |
| 0.6 | 24.71 [23.09, 26.32] | 26.30 [23.78, 28.82] | 26.14 [24.99, 27.29] | 30.19 [28.32, 32.06] |
| 0.8 | 18.31 [16.06, 20.56] | 18.37 [15.11, 21.63] | 19.17 [17.56, 20.79] | 22.96 [20.46, 25.45] |
| 1.0 | 12.32 [9.71, 14.93] | 11.39 [7.82, 14.96] | 12.56 [10.64, 14.47] | 16.05 [13.00, 19.09] |
|  |  |  |  |  |
| Score | **Estimated residual life expectancy [95% CI], 65 y** | | | |
| 0.0 | 25.06 [23.50, 26.63] | 30.13 [28.86, 31.40] | 27.53 [26.37, 28.70] | 30.60 [29.67, 31.52] |
| 0.2 | 19.41 [18.15, 20.68] | 24.72 [23.0, 26.47] | 21.35 [20.24, 22.46] | 25.77 [24.48, 27.06] |
| 0.4 | 14.11 [13.11, 15.11] | 17.54 [15.67, 19.41] | 15.09 [14.26, 15.92] | 19.27 [17.84, 20.71] |
| 0.6 | 9.75 [8.73, 10.77] | 11.14 [9.35, 12.93] | 9.93 [9.14, 10.72] | 13.18 [11.71, 14.65] |
| 0.8 | 6.37 [5.32, 7.42] | 6.51 [4.86, 8.17] | 6.01 [5.22, 6.79] | 8.36 [6.88, 9.85] |
| 1.0 | 3.89 [2.91, 4.87] | 3.50 [2.17, 4.84] | 3.27 [2.59, 3.96] | 4.86 [3.52, 6.21] |

Y=years; p=participants; HR=hazard ratio; CI=confidence intervals.

Models adjusted for ethnicity (white, non-white), working status (working, retired, other), deprivation (continuous), body mass index (continuous), sedentary time (continuous).

^a^ To make the HR interpretable the score was rescaled to measure 1 unit change using 0 to 100 score.

The continuous score should be interpreted alongside the coefficients reported in **Table S4.A**.
