# Supplementary material for: Healthy lifestyle and life expectancy in people with multimorbidity in the UK Biobank: A longitudinal cohort study
Source: PLoS Med. 2020 Sep 22;17(9):e1003332. doi: 10.1371/journal.pmed.1003332 (PMC7508366; doi:10.1371/journal.pmed.1003332)
Supplement: S11 Table — (DOCX) [file pmed.1003332.s016.docx]

# S11 Table: Survival using weighted score by cardiometabolic multimorbidity

| Healthy lifestyle category | With cardiometabolic multimorbidity | | Without cardiometabolic multimorbidity | |
| --- | --- | --- | --- | --- |
|  | **Men**  (n=2,838) | **Women**  (n=966) | **Men**  (n=215,990) | **Women**  (n=261,146) |
| No. deaths / No. participants | | | | |
| Very unhealthy | 42 / 288 | 13 / 96 | 1,123 / 19,807 | 543 / 16,445 |
| Unhealthy | 9 / 62 | 1 / 20 | 268 / 6,641 | 159 / 6,449 |
| Healthy | 137 / 1,087 | 32 / 344 | 1,988 / 65,079 | 1,343 / 84,431 |
| Very healthy | 165 / 1,401 | 30 / 506 | 2,880 / 124,463 | 2,273 / 153,821 |
|  |  |  |  |  |
| HR (95% CI) | | | | |
| Very unhealthy | 1 (Reference) | 1 (Reference) | 1 (Reference) | 1 (Reference) |
| Unhealthy | 1.05 (0.51, 2.16) | 0.47 (0.06, 3.63) | 0.71 (0.62, 0.82) | 0.77 (0.65, 0.92) |
| Healthy | 0.80 (0.56, 1.13) | 0.76 (0.39, 1.50) | 0.50 (0.46, 0.53) | 0.44 (0.40, 0.49) |
| Very healthy | 0.75 (0.53, 1.06) | 0.50 (0.25, 1.00) | 0.37 (0.35, 0.40) | 0.39 (0.35, 0.43) |
|  |  |  |  |  |
| Years of life gained [95% CI], 45 y | | | | |
| Very unhealthy | Reference | Reference | Reference | Reference |
| Unhealthy | -0.41 [-6.60, 5.77] | 6.25 [-8.60, 21.11] | 2.46 [1.43, 3.49] | 2.06 [0.64, 3.47] |
| Healthy | 1.94 [-1.29, 5.17] | 2.48 [-3.92, 8.87] | 5.13 [4.37, 5.90] | 6.18 [5.25, 7.11] |
| Very healthy | 2.53 [-0.80, 5.87] | 5.81 [-0.77, 12.39] | 7.17 [6.32, 8.02] | 6.93 [6.01, 7.85] |
|  |  |  |  |  |
| Years of life gained [95% CI], 65 y | | | | |
| Very unhealthy | Reference | Reference | Reference | Reference |
| Unhealthy | -0.32 [-5.07, 4.44] | 5.19 [-7.17, 17.55] | 2.09 [1.20, 2.98] | 1.80 [0.56, 3.05] |
| Healthy | 1.53 [-1.04, 4.10] | 2.05 [-3.26, 7.35] | 4.43 [3.74, 5.12] | 5.50 [4.66, 6.34] |
| Very healthy | 2.01 [-0.67, 4.69] | 4.82 [-0.71, 10.34] | 6.27 [5.49, 7.04] | 6.19 [5.35, 7.02] |

Y=years; p=participants; HR=hazard ratio; CI=confidence intervals; CVD=stroke, myocardial infarction, heart failure, angina or peripheral vascular disease.

Models adjusted for ethnicity (white, non-white), working status (working, retired, other), deprivation (continuous), body mass index (continuous), sedentary time (continuous).
