# Supplementary material for: Healthy lifestyle and life expectancy in people with multimorbidity in the UK Biobank: A longitudinal cohort study
Source: PLoS Med. 2020 Sep 22;17(9):e1003332. doi: 10.1371/journal.pmed.1003332 (PMC7508366; doi:10.1371/journal.pmed.1003332)
Supplement: S12 Table — (DOCX) [file pmed.1003332.s017.docx]

# S12 Table: Survival using individual lifestyle factor by cardiometabolic multimorbidity

| Healthy lifestyle factor | With cardiometabolic multimorbidity | | Without cardiometabolic multimorbidity | |
| --- | --- | --- | --- | --- |
|  | **Men**  (n=2,838) | **Women**  (n=966) | **Men**  (n=215,990) | **Women**  (n=261,146) |
| Regular physical activity | | | | |
| No - No. of deaths/participants | 233 / 1,1729 | 67 / 710 | 3,238 / 90,888 | 2,569 / 136,618 |
| Yes - No. of deaths/participants | 120 / 1,109 | 9 / 256 | 3,021 / 125,102 | 1,749 / 124,528 |
| HR (95% CI), Yes vs No (reference) | 0.81 (0.64, 1.02) | 0.39 (0.19, 0.79) | 0.74 (0.70, 0.78) | 0.82 (0.77, 0.88) |
| Years of life gained [95% CI], 45 y | 1.87 [-0.37, 4.11] | 6.34 [1.35, 11.33] | 2.19 [1.77, 2.62] | 1.27 [0.86, 1.69] |
| Years of life gained [95% CI], 65 y | 1.50 [-0.34, 3.35] | 5.22 [0.97, 9.48] | 1.95 [1.57, 2.34] | 1.15 [0.77, 1.52] |
|  |  |  |  |  |
| Smoking | | | | |
| Smoker - No. of deaths/participants | 51 / 350 | 14 / 116 | 1,391 / 26,448 | 702 / 22,894 |
| No current smoking - No. of deaths/participants | 302 / 2,488 | 62 / 850 | 4,868 / 189,542 | 3,616 / 238,252 |
| HR (95% CI), No vs Yes (reference) | 0.78 (0.57, 1.06) | 0.75 (0.40, 1.40) | 0.47 (0.44, 0.50) | 0.45 (0.41, 0.49) |
| Years of life gained [95% CI], 45 y | 2.16 [-0.74, 5.06] | 2.00 [-2.67, 6.67] | 5.51 [4.82, 6.21] | 5.87 [5.09, 6.65] |
| Years of life gained [95% CI], 65 y | 1.71 [-0.62, 4.03] | 1.61 [-2.19, 5.42] | 4.81 [4.18, 5.44] | 5.24 [4.54, 5.95] |
|  |  |  |  |  |
| Healthy diet | | | | |
| No - No. of deaths/participants | 218 / 1,723 | 45 / 522 | 4,438 / 148,538 | 2,441 / 147,477 |
| Yes - No. of deaths/participants | 135 / 1,115 | 31 / 444 | 1,821 / 67,452 | 1,877 / 113,669 |
| HR (95% CI), Yes vs No (reference) | 0.97 (0.78, 1.21) | 0.90 (0.56, 1.43) | 0.90 (0.85, 0.95) | 0.95 (0.90, 1.01) |
| Years of life gained [95% CI], 45 y | 0.25 [-1.66, 2.15] | 0.71 [-2.32, 3.75] | 0.76 [0.36, 1.16] | 0.31 [-0.09, 0.71] |
| Years of life gained [95% CI], 65 y | 0.20 [-1.33, 1.73] | 0.58 [-1.88, 3.03] | 0.67 [0.32, 1.03] | 0.28 [-0.09, 0.64] |
|  |  |  |  |  |
| Alcohol consumption | | | | |
| Excess - No. of deaths/participants | 119 / 983 | 5 / 82 | 3,194 / 111,195 | 980 / 68,164 |
| None/moderate - No. of deaths/participants | 234 / 1,855 | 71 / 884 | 3,065 / 104,795 | 3,338 / 192,982 |
| HR (95% CI), None/moderate vs Excess (reference) | 1.04 (0.83, 1.30) | 1.33 (0.54, 3.32) | 1.01 (0.96, 1.06) | 1.07 (1.00, 1.15) |
| Years of life gained [95% CI], 45 y | -0.32 [-2.28, 1.64] | -1.85 [-7.40, 3.71] | -0.05 [-0.41, 0.31] | -0.45 [-0.91, 0.02] |
| Years of life gained [95% CI], 65 y | -0.26 [-1.84, 1.32] | -1.49 [-6.01, 3.03] | -0.04 [-0.36, 0.28] | -0.40 [-0.82, 0.02] |

Y=years; p=participants; HR=hazard ratio; CI=confidence intervals; ref=reference; CVD=stroke, myocardial infarction, heart failure, angina or peripheral vascular disease.

Regular physical activity: ≥500 MET-minutes/week; Healthy diet: at least five portions of fruit and vegetables every day; None/moderate alcohol consumption: 0 to 14 units of alcohol a week.

Model adjusted for ethnicity (white, non-white), working status (working, retired, other), deprivation (continuous), body mass index (continuous), sedentary time (continuous) and all other healthy lifestyle factors. The reference for years of life gained is the same used for hazard ratio
