# Supplementary material for: Healthy lifestyle and life expectancy in people with multimorbidity in the UK Biobank: A longitudinal cohort study
Source: PLoS Med. 2020 Sep 22;17(9):e1003332. doi: 10.1371/journal.pmed.1003332 (PMC7508366; doi:10.1371/journal.pmed.1003332)
Supplement: S13 Table — (DOCX) [file pmed.1003332.s018.docx]

# S13 Table: Survival using number of healthy lifestyle risk factors (score 0-4) by multimorbidity

| Number of health lifestyle factors | With multimorbidity | | Without multimorbidity | |
| --- | --- | --- | --- | --- |
|  | **Men**  (n=43,448) | **Women**  (n=50,298) | **Men**  (n=175,380) | **Women**  (n=211,814) |
| No. deaths / No. participants | | | | |
| Zero (Unhealthiest) | 161 / 1,364 | 46 / 639 | 271 / 5,312 | 73 / 2,910 |
| One | 567 / 8,199 | 220 / 5,724 | 938 / 31,244 | 375 / 22,942 |
| Two | 974 / 16,761 | 583 / 18,015 | 1,477 / 68,307 | 936 / 72,635 |
| Three | 732 / 12,964 | 517 / 18,189 | 1,005 / 53,721 | 1,007 / 76,900 |
| Four (Healthiest) | 185 / 4,160 | 193 / 7,731 | 302 / 16,796 | 444 / 36,427 |
|  |  |  |  |  |
| HR (95% CI) | | | | |
| Zero (Unhealthiest) | 1 (Reference) | 1 (Reference) | 1 (Reference) | 1 (Reference) |
| One | 0.55 (0.46, 0.66) | 0.48 (0.35, 0.66) | 0.57 (0.49, 0.65) | 0.55 (0.43, 0.71) |
| Two | 0.46 (0.39, 0.55) | 0.38 (0.28, 0.52) | 0.40 (0.35, 0.46) | 0.40 (0.32, 0.51) |
| Three | 0.43 (0.36, 0.52) | 0.32 (0.24, 0.44) | 0.33 (0.29, 0.38) | 0.38 (0.30, 0.48) |
| Four (Healthiest) | 0.33 (0.27, 0.41) | 0.29 (0.21, 0.40) | 0.30 (0.26, 0.36) | 0.34 (0.26, 0.43) |
|  |  |  |  |  |
| Years of life gained [95% CI], 45 y | | | | |
| Zero (Unhealthiest) | Reference | Reference | Reference | Reference |
| One | 4.80 [3.11, 6.48] | 6.51 [3.43, 9.60] | 4.19 [2.99, 5.40] | 4.56 [2.50, 6.63] |
| Two | 6.24 [4.50, 7.99] | 8.37 [5.38, 11.36] | 6.70 [5.37, 8.04] | 6.68 [4.65, 8.70] |
| Three | 6.75 [4.93, 8.56] | 9.63 [6.61, 12.64] | 8.07 [6.63, 9.50] | 7.09 [5.06, 9.11] |
| Four (Healthiest) | 8.78 [6.59, 10.97] | 10.37 [7.23, 13.51] | 8.64 [7.04, 10.25] | 7.82 [5.73, 9.90] |
|  |  |  |  |  |
| Years of life gained [95% CI], 65 y | | | | |
| Zero (Unhealthiest) | Reference | Reference | Reference | Reference |
| One | 3.84 [2.48, 5.21] | 5.44 [2.90, 7.98] | 3.61 [2.56, 4.66] | 4.06 [2.23, 5.88] |
| Two | 5.06 [3.62, 6.49] | 7.04 [4.57, 9.51] | 5.86 [4.67, 7.06] | 5.97 [4.18, 7.76] |
| Three | 5.49 [3.99, 6.99] | 8.13 [5.63, 10.63] | 7.10 [5.82, 8.39] | 6.35 [4.56, 8.14] |
| Four (Healthiest) | 7.25 [5.40, 9.10] | 8.78 [6.17, 11.38] | 7.63 [6.18, 9.08] | 7.02 [5.17, 8.87] |

Y=years; p=participants; HR=hazard ratio; CI=confidence intervals.

Models adjusted for ethnicity (white, non-white), working status (working, retired, other), deprivation (continuous), body mass index (continuous), sedentary time (continuous).
