# Supplementary material for: Healthy lifestyle and life expectancy in people with multimorbidity in the UK Biobank: A longitudinal cohort study
Source: PLoS Med. 2020 Sep 22;17(9):e1003332. doi: 10.1371/journal.pmed.1003332 (PMC7508366; doi:10.1371/journal.pmed.1003332)
Supplement: S14 Table — (DOCX) [file pmed.1003332.s019.docx]

# S14 Table: Survival using number of healthy lifestyle risk factors (score 0-4) by cardiometabolic multimorbidity

| Number of health lifestyle factors | With cardiometabolic multimorbidity | | Without cardiometabolic multimorbidity | |
| --- | --- | --- | --- | --- |
|  | **Men**  (n=2,838) | **Women**  (n=966) | **Men**  (n=215,990) | **Women**  (n=261,146) |
| No. deaths / No. participants | | | | |
| Zero (Unhealthiest) | 14 / 70 | 0 / 4 | 418 / 6,606 | 119 / 3,545 |
| One | 60 / 476 | 14 / 102 | 1,445 / 38,967 | 581 / 28,564 |
| Two | 134 / 1,099 | 32 / 359 | 2,317 / 83,969 | 1,487 / 90,291 |
| Three | 117 / 879 | 25 / 390 | 1,620 / 65,806 | 1,499 / 94,699 |
| Four (Healthiest) | 28 / 314 | 5 / 111 | 459 / 20,642 | 632 / 44,047 |
|  |  |  |  |  |
| HR (95% CI) | | | | |
| Zero (Unhealthiest) | 1 (Reference) | 1 (Reference) | 1 (Reference) | 1 (Reference) |
| One | 0.63 (0.35, 1.14) | * | 0.56 (0.50, 0.62) | 0.51 (0.42, 0.63) |
| Two | 0.62 (0.35, 1.07) | * | 0.41 (0.37, 0.46) | 0.39 (0.32, 0.47) |
| Three | 0.66 (0.38, 1.16) | * | 0.35 (0.32, 0.40) | 0.35 (0.29, 0.42) |
| Four (Healthiest) | 0.42 (0.22, 0.80) | * | 0.31 (0.27, 0.35) | 0.31 (0.25, 0.38) |
|  |  |  |  |  |
| Years of life gained [95% CI], 45 y | | | | |
| Zero (Unhealthiest) | Reference | Reference | Reference | Reference |
| One | 3.91 [-1.61, 9.44] | * | 4.28 [3.32, 5.24] | 5.23 [3.53, 6.93] |
| Two | 4.15 [-1.18, 9.47] | * | 6.48 [5.43, 7.54] | 7.25 [5.59, 8.92] |
| Three | 3.53 [-1.70, 8.76] | * | 7.60 [6.47, 8.72] | 7.94 [6.27, 9.62] |
| Four (Healthiest) | 7.52 [0.69, 14.35] | * | 8.55 [7.26, 9.84] | 8.70 [6.70, 10.43] |
|  |  |  |  |  |
| Years of life gained [95% CI], 65 y | | | | |
| Zero (Unhealthiest) | Reference | Reference | Reference | Reference |
| One | 3.00 [-1.26, 7.27] | * | 3.63 [2.80, 4.45] | 4.58 [3.11, 6.06] |
| Two | 3.19 [-0.92, 7.30] | * | 5.57 [4.65, 6.50] | 6.40 [4.95, 7.85] |
| Three | 2.70 [-1.30, 6.70] | * | 6.58 [5.59, 7.57] | 7.03 [5.56, 8.49] |
| Four (Healthiest) | 5.96 [0.47, 11.45] | * | 7.44 [6.29, 8.59] | 7.71 [6.20, 9.22] |

Y=years; p=participants; HR=hazard ratio; CI=confidence intervals. CVD=stroke, myocardial infarction, heart failure, angina or peripheral vascular disease.

* Number of events in the reference category was 0, unable to predict survival.

Models adjusted for ethnicity (white, non-white), working status (working, retired, other), deprivation (continuous), body mass index (continuous), sedentary time (continuous).
