# Supplementary material for: Healthy lifestyle and life expectancy in people with multimorbidity in the UK Biobank: A longitudinal cohort study
Source: PLoS Med. 2020 Sep 22;17(9):e1003332. doi: 10.1371/journal.pmed.1003332 (PMC7508366; doi:10.1371/journal.pmed.1003332)
Supplement: S15 Table — (DOCX) [file pmed.1003332.s020.docx]

# S15 Table: Survival in the matched cohort

| Healthy lifestyle category | Men | | Women | |
| --- | --- | --- | --- | --- |
|  | Non matched cohort (n=175,380) | Matched cohort  (n=33,752) | Non matched cohort (n=211,814) | Matched cohort  (n=40,261) |
| No. deaths / No. participants | | | | |
| Very unhealthy | 744 / 15,991 | 225 / 3,508 | 331 / 12,823 | 90 / 2,729 |
| Unhealthy | 180 / 5,553 | 52 / 1,118 | 116 / 5,328 | 26 / 1,011 |
| Healthy | 1,183 / 51,127 | 290 / 10,222 | 831 / 67,190 | 215 / 13,386 |
| Very healthy | 1,886 / 102,709 | 476 / 18,904 | 1,557 / 126,473 | 368 / 23,135 |
| HR (95% CI) | | | | |
| Very unhealthy | Reference (1) | Reference (1) | Reference (1) | Reference (1) |
| Unhealthy | 0.69 (0.58, 0.81) | 0.70 (0.52, 0.94) | 0.84 (0.68, 1.04) | 0.80 (0.51, 1.23) |
| Healthy | 0.46 (0.42, 0.51) | 0.41 (0.34, 0.49) | 0.44 (0.38, 0.50) | 0.45 (0.35, 0.57) |
| Very healthy | 0.35 (0.32, 0.39) | 0.35 (0.30, 0.42) | 0.40 (0.36, 0.46) | 0.43 (0.34, 0.54) |
| Years of life gained [95% CI], 45 y | | | | |
| Very unhealthy | Reference | Reference | Reference | Reference |
| Unhealthy | 2.77 [1.49, 4.05] | 2.95 [0.37, 5.53] | 1.34 [-0.31, 2.99] | 1.74 [-1.62, 5.09] |
| Healthy | 5.66 [4.65, 6.66] | 7.16 [5.26, 9.07] | 6.03 [4.90, 7.15] | 5.79 [3.62, 7.96] |
| Very healthy | 7.56 [6.47, 8.64] | 8.20 [6.31, 10.08] | 6.49 [5.39, 7.59] | 6.03 [3.91, 8.15] |
| Years of life gained [95% CI], 65 y | | | | |
| Very unhealthy | Reference | Reference | Reference | Reference |
| Unhealthy | 2.39 [1.26, 3.52] | 2.54 [0.29, 4.79] | 1.19 [-0.28, 2.66] | 1.55 [-1.45, 4.54] |
| Healthy | 4.96 [4.05, 5.88] | 6.28 [4.57, 7.99] | 5.42 [4.40, 6.45] | 5.22 [3.24, 7.19] |
| Very healthy | 6.70 [5.69, 7.70] | 7.21 [5.51, 8.91] | 5.85 [4.84, 6.85] | 5.44 [3.51, 7.37] |

Y=years; p=participants; HR=hazard ratio; CI=confidence intervals.

Estimates are shown for people without multimorbidity who were matched to those with multimorbidity. Models adjusted for ethnicity (white, non-white), working status (working, retired, other), deprivation (continuous), body mass index (continuous), sedentary time (continuous).
