# Supplementary material for: Healthy lifestyle and life expectancy in people with multimorbidity in the UK Biobank: A longitudinal cohort study
Source: PLoS Med. 2020 Sep 22;17(9):e1003332. doi: 10.1371/journal.pmed.1003332 (PMC7508366; doi:10.1371/journal.pmed.1003332)
Supplement: S1 Fig — (DOCX) [file pmed.1003332.s021.docx]

# **S1 Fig:** Flow chart of participants included in the study

Baseline assessment

N=502,629

Participants with missing healthy lifestyle factors

(Physical activity, smoking, diet, alcohol, body mass index)

n = 16,503 (3.3%)

Participants aged less than 45 years n = 30 (0.01%)

Participants who died in less than 2 years

n = 2,516 (0.5%)

Participants who withdrew

n = 91 (0.02%)

Participants with missing covariate data

(Ethnicity, deprivation, sedentary behaviour)

n = 2,549 (0.51%)

Main analysis: complete case

N = 480,940 (95.7%)

Sensitivity analysis: imputed missing data

(Smoking, diet, alcohol, body mass index, ethnicity, deprivation, sedentary behaviour)

N = 488,475 (97.2%)
