# Supplementary material for: Healthy lifestyle and life expectancy in people with multimorbidity in the UK Biobank: A longitudinal cohort study
Source: PLoS Med. 2020 Sep 22;17(9):e1003332. doi: 10.1371/journal.pmed.1003332 (PMC7508366; doi:10.1371/journal.pmed.1003332)
Supplement: S3 Fig — (DOCX) [file pmed.1003332.s023.docx]

# S3 Fig: Estimated residual life expectancy using the continuous weighted lifestyle score obtained from a random one third of the population

Models adjusted for ethnicity (white, non-white), working status (working, retired, other), deprivation (continuous), body mass index (continuous), sedentary time (continuous).

The continuous score should be interpreted alongside the coefficients reported in **Table S4.A**.
