# Supplementary material for: Healthy lifestyle and life expectancy in people with multimorbidity in the UK Biobank: A longitudinal cohort study
Source: PLoS Med. 2020 Sep 22;17(9):e1003332. doi: 10.1371/journal.pmed.1003332 (PMC7508366; doi:10.1371/journal.pmed.1003332)
Supplement: S1 Checklist — STROBE, Strengthening the Reporting of Observational Studies in Epidemiology (DOCX) [file pmed.1003332.s026.docx]

# S1 Checklist: Strengthening the Reporting of Observational Studies in Epidemiology (STROBE)

|  | Item No | Recommendation |  |
| --- | --- | --- | --- |
| **Title and abstract** | 1 | (*a*) Indicate the study’s design with a commonly used term in the title or the abstract | Abstract, methods and findings |
|  |  | (*b*) Provide in the abstract an informative and balanced summary of what was done and what was found | Abstract, methods and findings |
| Introduction | | |  |
| Background/rationale | 2 | Explain the scientific background and rationale for the investigation being reported | Introduction paragraph 1 & 2 |
| Objectives | 3 | State specific objectives, including any prespecified hypotheses | Introduction paragraph 3 |
| Methods | | |  |
| Study design | 4 | Present key elements of study design early in the paper | Methods, Study Population |
| Setting | 5 | Describe the setting, locations, and relevant dates, including periods of recruitment, exposure, follow-up, and data collection | Methods, Study Population |
| Participants | 6 | (*a*) *Cohort study*—Give the eligibility criteria, and the sources and methods of selection of participants. Describe methods of follow-up | Methods, Study Population |
|  |  | (*b*) *Cohort study*—For matched studies, give matching criteria and number of exposed and unexposed | NA |
| Variables | 7 | Clearly define all outcomes, exposures, predictors, potential confounders, and effect modifiers. Give diagnostic criteria, if applicable | Methods, Multimorbidity, Mortality, Healthy lifestyle, Confounders |
| Data sources/ measurement | 8* | For each variable of interest, give sources of data and details of methods of assessment (measurement). Describe comparability of assessment methods if there is more than one group | Methods, Multimorbidity, Mortality, Healthy lifestyle, Confounders |
| Bias | 9 | Describe any efforts to address potential sources of bias | Methods, Study Population, Statistical Analysis paragraph 3 |
| Study size | 10 | Explain how the study size was arrived at | Supporting Information, S1 Figure |
| Quantitative variables | 11 | Explain how quantitative variables were handled in the analyses. If applicable, describe which groupings were chosen and why | Methods, Multimorbidity, Healthy lifestyle, Statistical analysis |
| Statistical methods | 12 | (*a*) Describe all statistical methods, including those used to control for confounding | Methods, Statistical analysis |
|  |  | (*b*) Describe any methods used to examine subgroups and interactions | NA |
|  |  | (*c*) Explain how missing data were addressed | Methods, Statistical analysis paragraph 3. Supporting Information S3 Methods |
|  |  | (*d*) *Cohort study*—If applicable, explain how loss to follow-up was addressed | NA |
|  |  | (*e*) Describe any sensitivity analyses | Methods, Statistical analysis paragraph 3 |
| Results | | |  |
| Participants | 13* | (a) Report numbers of individuals at each stage of study—e.g. numbers potentially eligible, examined for eligibility, confirmed eligible, included in the study, completing follow-up, and analysed | Methods, Study Population |
|  |  | (b) Give reasons for non-participation at each stage | Methods, Study Population |
|  |  | (c) Consider use of a flow diagram | Supporting Information, S1 Figure |
| Descriptive data | 14* | (a) Give characteristics of study participants (e.g. demographic, clinical, social) and information on exposures and potential confounders | Results, Baseline characteristics paragraph 1 & 2 |
|  |  | (b) Indicate number of participants with missing data for each variable of interest | Supporting Information S3 Methods |
|  |  | (c) *Cohort study*—Summarise follow-up time (e.g., average and total amount) | Results, Healthy lifestyle, paragraph 1 |
| Outcome data | 15* | *Cohort study*—Report numbers of outcome events or summary measures over time | Results, Healthy lifestyle, paragraph 1 |
| Main results | 16 | (*a*) Give unadjusted estimates and, if applicable, confounder-adjusted estimates and their precision (eg, 95% confidence interval). Make clear which confounders were adjusted for and why they were included | Results, Healthy lifestyle, paragraph 1 & 2, Individual lifestyle factor paragraph 1 & 2 |
|  |  | (*b*) Report category boundaries when continuous variables were categorized | S2 Methods |
|  |  | (*c*) If relevant, consider translating estimates of relative risk into absolute risk for a meaningful time period | Results |
| Other analyses | 17 | Report other analyses done—e.g. analyses of subgroups and interactions, and sensitivity analyses | Results, Sensitivity analyses |
| Discussion | | |  |
| Key results | 18 | Summarise key results with reference to study objectives | Discussion paragraph 1 |
| Limitations | 19 | Discuss limitations of the study, taking into account sources of potential bias or imprecision. Discuss both direction and magnitude of any potential bias | Discussion paragraph 6 |
| Interpretation | 20 | Give a cautious overall interpretation of results considering objectives, limitations, multiplicity of analyses, results from similar studies, and other relevant evidence | Discussion paragraph 3-6 |
| Generalisability | 21 | Discuss the generalisability (external validity) of the study results | Discussion paragraph 7 |
| Other information | | |  |
| Funding | 22 | Give the source of funding and the role of the funders for the present study and, if applicable, for the original study on which the present article is based. | Published alongside the manuscript |
