# Supplementary material for: Healthy lifestyle and life expectancy in people with multimorbidity in the UK Biobank: A longitudinal cohort study
Source: PLoS Med. 2020 Sep 22;17(9):e1003332. doi: 10.1371/journal.pmed.1003332 (PMC7508366; doi:10.1371/journal.pmed.1003332)
Supplement: Attachment — Submitted filename: R1_Response to reviewers.pdf [file pmed.1003332.s027.pdf]

Requests from the editors	
**Comments**	**Response**
1. We advise you to carefully respond to all of the reviewer comments, as this will be taken into consideration when deciding whether to accept your manuscript for publication. Most importantly, please address the concerns raised by reviewers #1 and #2 regarding the statistical methodology employed in your study.	We have answered point-by-point to all reviewers' comments and modified the paper accordingly. We have performed all extra analyses and amended the manuscript in line with the suggestions.
2. Please remove the word "prospective" from the title (we believe that your paper reports a retrospective analysis of a prospectively gathered dataset).	The word has been removed.
3. Abstract:<br>a. Please include the study design, population demographics (eg. age range, sex), and dates during which the study data were collected.<br>b. In the last sentence of the Abstract Methods and Findings section, please describe the main limitation(s) of the study's methodology.<br>c. In the Methods and Findings subsection of your abstract, please summarize the factors adjusted for.<br>d. In the Conclusions subsection of your abstract, please write "... healthier lifestyle was associated ...".	We have modified the abstract as suggested.
4. Please use the "Vancouver" style for reference formatting, and see our website for other reference guidelines<br>a. Citations in the main text should come before punctuation, e.g., "... multimorbidity measures [1,21,24].<br>b. In your reference list, please abbreviate journal names consistently (e.g., "PLoS Med.").	We have modified the style of the reference as requested.
5. In the Abstract and throughout the main text, please include p values alongside CIs for your numerical data.	We have added p-values for all CIs.
6. Please avoid use of the term "effect" when describing your findings of association.	We have removed this term when we referred to associations.

Requests from the editors	
**Comments**	**Response**
7. Please remove the data, funding, author contributions, and competing interests statements from page 18 – these are published from corresponding fields on the submission form.	Removed.
8. In your STROBE checklist, please use section and paragraph numbers, rather than page numbers. Please also add the following statement, or similar, to the Methods: "This study is reported as per the Strengthening the Reporting of Observational Studies in Epidemiology (STROBE) guideline (S1 Checklist)."	We have amended the checklist and the methods paragraph as indicated.
9. Please include line numbers throughout your manuscript.	Added.
10. We believe you refer to the UK Biobank ethics approval in your methods section. Please also mention the ethics situation for the present study (e.g., cite approval by local IRB).	We have clarified in the methods section that the ethics approval was generic for UK Biobank and not specific from a local IRB, as this was not required.
11. Early in the Results section, please write "fewer participants".	Modified as suggested.

Reviewer #1	
**Comments**	**Response**
I confine my remarks to statistical aspects of this paper.<br><br>There is one major problem that, unfortunately, means that all the analysis has to be redone.<br><br>The authors have categorized every continuous variable. This is a mistake. Categorizing continuous variable increases both type I and type II error, it also introduces a kind of magical thinking - i..e. that something amazing happens right at the cutpoint. Frank Harrell, in *Regression Modelling Strategies* listed 11 problems that categorizing independent variables can cause and summed up "nothing could be more disastrous". I wrote a blog post demonstrating some of these problems graphically https://eur03.safelinks.protection.outlook.com/?url=https%3A%2F%2Fmedium.com%2F%40peterflom%2Fwhat- happens-when-we-categorize-an-independent-variable-in-regression-77d4c5862b6c&data=02%7C01%7Cyc244%40leicester.ac.uk%7C90a7a87d5ea541d5103d08d7f5c29f12%7Caebecd6a31d44b0195ce8274afe853d9%7C0%7C0%7C637248088550784122&sdata=47f2kLBDB04jgYDHbK7HUjqnZUlWOyKvXwrqk3MuIuc%3D&reserved=0<br><br>All the variables should be left continuous. Splines can be used to look for nonlinearities.<br><br>These changes would affect all of the subsequent write up, so I will wait for a revision to do a review of those parts.<br><br>Peter Flom	Thank you Dr Flom for your comment and for your interesting blog.<br><br>We are actually very strong supporter of the use of continuous variables (whenever possible) instead of categorising them, as well as of their transformation using splines where appropriate. We recognise in this regard the past and continuous efforts of Frank Harrell, Martin Bland, Doug Altman, Andy Wickers and Stephen Senn, who are among those who have most warned against the use of "dichotimisation". A very useful and updated discussion has been recently added as a wiki on Frank Harrell's blog (https://discourse.datmethods.org/t/categorizing-continuous-variables/3402).<br><br>In this view, while preparing our analytical plan, we tried to balance statistical points with public health messages. In fact, we used categories to define whether a risk factor was under control (using thresholds reported in public health guidelines); then we constructed an overall continuous weighted score; lastly, this score was divided in 4 groups (from very unhealthy to very healthy) in the attempt to enhance the public health message. Our decision seemed to have been only partly appreciated by other reviewers, as they suggest that the public health message would be even stronger if we complement our analyses with a new analysis using the simple sum of the risk factors, i.e., 0/1 if each "healthy condition" is satisfied [1 – not a smoker; 0 – a smoker], so that the sum of 4 indicates a "healthy" lifestyle. We agree with this point as well, which is in line with the vast majority of previous literature about healthy lifestyle scores and, more importantly, with current public health guidelines about healthy lifestyle (i.e., <14 units/week alcohol; ≥500 MET-minutes/week of physical activity, etc…all using thresholds).<br><br>While preparing a revision of this paper, we were therefore between two apparently contrasting, but rather complementary, views. We have re-analysed all data using both a score (0 to 4), and a continuous score obtained from continuous variables, as you suggested.<br><br>The analytical steps, reported also in the S4 Methods, were:<br><br>1 – The 3 continuous components of the score (physical activity in metabolic equivalents [METs]; units of alcohol per week; and daily portions of fruit/vegetables] were modelled as continuous variables instead of using above/below guidelines-defined thresholds.<br><br>2 – Two survival models (with time to death as outcome) were then compared:

Reviewer #1
**Response**

(a) with 3 continuous linear variables + smoking (available as former/current/never);
(b) with transformation of the 3 variables using restricted cubic spline with knots at the 10th, 30th, 50th, 70th, 90th centile of their distributions + smoking

The models (a) and (b) were then compared with a (partial) likelihood ratio test, indicating a "statistically significant" ($p<0.05$) difference between the two. In particular, both the AIC and the BIC indicated that the model with continuous non-linear variables was "better" (lower values).

3 – We then run model (b) and estimated the linear predictor (log hazard ratio), which is the individual "lifestyle score". For easy of interpretation (and comparability with the score we calculated in the original submission), we rescaled the score from 0 to 1.

We made predictions for 0.1 unit increase of score from 0 to 1 to plot the mean estimated residual life vs score and, given the large computational time, estimated uncertainties (i.e., 95% CI) at values of score of 0, 0.2, 0.4, 0.6, 0.8, and 1. To assess the robustness of the results, we also estimated the score in a random 1/3 of the sample and applied it to the remaining 2/3; and recalculated the score after imputing missing data.

In re-organising the paper, we have kept our previous analyses and added the extra analyses required by the reviewers at this revision stage. The overall new structure of results is shown in S2 Table.

The results of the analyses using the continuous score developed following the steps reported above are shown in S2-S4 Figures and S8-S10 Tables. We wish to highlight some points related to using this score. The association between the score and the estimated residual life within each group (participants with multimorbidity; participants without multimorbidity) can only be interpreted alongside the coefficients (including the spline ones) obtained from the regression model, as we have underlined when reporting these results. We believe that this could make the interpretation of the results difficult for the reader without expertise in statistical modelling, and probably it would be required to develop a nomogram/app to enhance interpretation. However, we believe that this implies a significant shift in the goal of the paper, where our main question was essentially aetiological rather than prognostic; in the latter case, in fact, reporting the coefficients alongside other steps (i.e., bootstrap, shrinkage, etc…) and developing a nomogram/app is the standard procedure (TRIPOD guidelines).

On the other hand, we recognise that using this continuous score still allows a straightforward interpretation of the differences between groups, i.e. comparing participants with and without multimorbidity. In fact, regardless of the value of the score, the estimated residual life is very similar (S2-S4 Figures and S8-S10 Tables), which is ultimately the main clinical/public health message of the paper.

Alternatively, your suggestion may be implemented in a different way: we may still use "useful" public health thresholds to define the score (so that a higher score indicates a healthier lifestyle) but, instead of creating 4 groups (from very unhealthy to very healthy) we used the continuous score. The advantage of this approach, compared to what we described just above, is that not only between-group but also within group association between the score and life expectancy have a straightforward interpretation. We have reported the results using this alternative approach in the **Table A** below.

We understand that, when the statistical approach requires a more complicated modelling than usual, there is not a single "correct" answer and decisions should be taken. We therefore thank you again for your suggestions and are happy to consider any further suggestion to ensure both statistical correctness and a clear public health message.

Many thanks for your suggestions and your time to review our manuscript.

Table A. Survival using the categories (dichotomised values) and then continuously modelling weighted score

Healthy lifestyle continuous weighted score	With multimorbidity		Without multimorbidity	
	**Men** (n=43,448)	**Women** (n=50,298)	**Men** (n=175,380)	**Women** (n=211,814)
**Score**	**Estimated residual life expectancy [95% CI], 45 y**			
0.0	32.82 [31.54, 34.11]	37.24 [35.11, 39.37]	34.83 [33.84, 35.81]	40.04 [38.48, 42.61]
0.2	34.43 [33.18, 35.68]	39.27 [37.28, 41.26]	36.67 [35.66, 37.68]	41.74 [40.27, 43.22]
0.4	36.05 [34.79, 37.30]	41.24 [39.37, 43.10]	38.53 [37.46, 39.59]	43.38 [41.99, 44.77]
0.6	37.67 [36.37, 38.97]	43.10 [41.34, 44.86]	40.38 [39.25, 41.51]	44.93 [43.62, 46.24]
0.8	39.28 [37.90, 40.67]	44.82 [43.15, 46.49]	42.20 [41.02, 43.39]	46.36 [45.12, 47.60]
1.0	40.87 [39.39, 42.35]	46.39 [44.80, 47.98]	43.96 [42.73, 45.18]	47.66 [46.49, 48.82]
**Score**	**Estimated residual life expectancy [95% CI], 45 y**			
0.0	15.51 [14.36, 16.65]	19.52 [17.50, 21.54]	16.53 [15.59, 17.47]	21.33 [19.81, 22.85]
0.2	16.77 [15.60, 17.94]	21.22 [19.27, 23.17]	18.09 [17.09, 19.09]	22.84 [21.38, 24.31]
0.4	18.08 [16.86, 19.30]	22.90 [21.03, 24.76]	19.71 [18.64, 20.78]	24.31 [22.91, 25.71]
0.6	19.43 [18.14, 20.73]	24.50 [22.73, 26.27]	21.36 [20.22, 22.50]	25.71 [24.39, 27.03]
0.8	20.81 [19.43, 22.18]	26.00 [24.33, 27.67]	23.01 [21.82, 24.21]	27.02 [25.77, 28.26]
1.0	22.18 [20.72, 23.64]	27.37 [25.80, 28.93]	24.62 [23.40, 25.85]	28.20 [27.04, 29.37]

Y=years; p=participants; HR=hazard ratio; CI=confidence intervals.
Models adjusted for ethnicity (white, non-white), working status (working, retired, other), deprivation (continuous), body mass index (continuous), sedentary time (continuous).

As the score was created using categorical variables the greater the score, the better the lifestyle.

Reviewer #2	
**Comments**	**Response**
This paper reports data on lifestyle and life expectancy in multimorbidity from the UK Biobank. This is an important topic and the dataset is sufficiently large to address the study question. I have the following comments for the author to consider:<br><br>1. The design is likely to introduce reverse causation bias in particularly because the authors have chosen a very broad definition for multimorbidity (any two or more of the 36 health conditions which vary in terms of severity). For example, a multimorbidity case with 2+ severe and disabling diseases may limited ability to exercise unlike another multimorbidity case with 2 mild health conditions; the association of physical inactivity with life expectancy will be overestimated in this case as the baseline difference in mortality risk between the two cases is not accounted for (ie the participant with 2+ severe conditions has a higher risk of dying independently of physical activity). The authors' attempt to reduce this kind of bias by excluding the first years of follow-up is a good but only partial solution. For this reason, I suggest they run a sensitivity analysis using a more homogeneous definition for multimorbidity - e.g. by looking the associations of lifestyle factors &score with life expectancy in participants with cardiometabolic multimorbidity (ie a combination of cvd and diabetes).	Many thanks.<br><br>In the revised paper, we have performed a sensitivity analysis using the more homogenous definition of multimorbidity limited to cardiometabolic conditions. We defined cardiometabolic multimorbidity as diabetes + CVD (stroke, myocardial infarction, heart failure, angina or peripheral vascular disease).<br><br>The results for the overall weighted score, the individual risk factors, and the score using the sum of the risk factors (question 3) are reported in S11, S12, and S14 Table, respectively.<br><br>These analyses highlighted the very few number of participants and events (particularly in women) which hampered a straightforward comparisons with findings using multimorbidity defined as 2+ conditions, yet for the individual risk factors the results were qualitatively similar. Therefore, we highlighted in the discussion [lines 475-482] that further research is required to explore whether a lifestyle factors and a healthier lifestyle is differently associated with life expectancy in relation to the types of multimorbidity.
2. The description of multimorbidity definition seems insufficient. It remains unclear how the authors decided which 36 chronic conditions they included in the definition. Why 36 rather than some other quantity and why these specific diseases? Is this a new definition or used also previously? Multimorbidity is a key variable in this paper, so the rationale for the definition should be clear.	We have better clarified the criteria used to define the 36 conditions, also highlighting that these conditions have been also considered in previous studies [lines 169-176], including ours (Ref. 8 and 25).<br><br>In particular, the Lancet manuscript we refer to in our paper (Ref. 1) is largely based on other two studies (Ref. 26 and 27) to define an initial set of comorbidities. Then, using clinical judgment, the Authors extracted a list of 40 conditions.<br><br>In our paper, we did not include some of them as they were part of the statistical modelling (i.e., alcohol is used for the definition of the lifestyle). However, we included other conditions which we deemed clinically relevant: anaemia, meningitis, tuberculosis and vestibular disorders.

Reviewer #2	
**Comments**	**Response**
3. The authors use a weighted lifestyle score which may introduce circularity bias. To obtain the weights, the authors first compute beta coefficients for each dichotomised lifestyle factor-mortality association. Then they construct a weighted lifestyle score by taking the sum of dichotomised lifestyle factors multiplied by the beta coefficient obtained from the mortality analysis. With this weighted lifestyle score, they estimate differences in life expectancy between those with higher and lower weighted lifestyle score - the main study question. These differences are expected because the weights for the exposure were based on information (ie mortality) from the outcome (life expectancy) - hence the circularity. I suggest that the authors run complementary analyses using a simple sum of dichotomised lifestyle factors as the exposure (range from 0 to 4). Unike the weighted lifestyle score, this indicator will allow comparison of the present findings to those from other studies in the field and it is not subject to circularity.	We completely agree with this point. Indeed, while planning our study, we considered this possibility which seems rarely mentioned in the literature when a score is created using this approach. We therefore accounted for this bias in the sensitivity analysis that estimated the score in a random 1/3 and applied it in the remaining 2/3. This is certainly not as perfect as using an externally developed score but it is a common procedure, for example, for an internal validation of a score (split sample approach).<br><br>However, we also completely agree that the proposed solution of a complementary analysis is not subject to this bias and would facilitate the comparisons with other studies. We have therefore complemented our analysis with a new one using a score ranging from 0 to 4.<br><br>The results are shown in S13 Table (for multimorbidity 2+) and S14 (for cardiometabolic multimorbidity). The years of life gained were slightly greater comparing heathiest vs unhealthiest groups in this analysis vs the analysis using the original weighted score [lines 348-351], but were on overall largely confirmed. Interestingly, using this approach our results are very well in line with previous evidence using a similar approach [lines 402-408].<br><br>About cardiometabolic multimorbidity, the limitation reported in the previous answer (very few participants) limited also the analyses using the score 0-4 (in some cases there were no events, S14 Table).
4. The authors have previously published on physical activity and life expectancy in multimorbidity using UK Biobank (BMC Med 2019) - this study should be noted in the introduction. The same in the description of the assessment of physical activity in this paper - did the authors use the same operationalisation? Are the findings on physical activity and life expectancy the same as in the previous paper?	We have now mentioned our paper in the introduction [lines 119-121].<br><br>In both papers, physical activity was considered but there are important differences. In the BMC Med analysis, we focused only on physical activity assessed from a self-reported questionnaire and objectively using accelerometer data: the goal was to evaluate its relevance, regardless of the definition/assessment used, on life expectancy.<br><br>In terms of estimating the METs, the approach is the same following the standardised methods reported in Ref. 33. However, the operationalisation was different: in the BMC paper, we considered a larger spectrum of variation of physical activity to investigate, from an aetiological perspective, the presence of a gradient in the association with life expectancy. In the current manuscript, conversely, we focused only on a single threshold (in line with current guidelines about healthy lifestyle and physical activity) as we consider it as one of the component of a more general score. Therefore, a direct comparison is not possible although in both analyses physical activity is associated with a longer survival.

Reviewer #2	
**Comments**	**Response**
5. Further details are needed on how winsorizing was done as there are many options.	We capped at 16 hrs for sleep, this has now been clarified in the text.
6. How the cut points for 'very unhealthy', 'unhealthy' etc for the lifestyle score categories were chosen?	We defined thresholds at 25th, 50th, and 75th centile of the distribution of the score to create the 4 groups [lines 244-246].
7. I am surprised by the prevalence of chronic conditions. Why cancer is more common than diabetes in men? Why cancer is more common than depression in women? Are there figures correct; sat least, they seem not to correspond to those observed in the general population. If correct, some discussion is needed on the reliability of measuring diseases using self-reports in the UK Biobank.	We agree that these results are surprising but they are indeed correct. The reason is possibly the limited representativeness of UK Biobank and certainly there is a "healthy volunteers" effect (Ref. 38). This may be the main reason, rather than bias in self-reports, of the observed prevalences.<br><br>We have further commented on the generalisability of UK Biobank in the revised paper [lines 442-452]. Please see also answer to question 10.
8. Discussion, first para. Two main findings are described. However, I do not think the comparison of lifestyle score vs multimorbidity in terms of which is more strongly associated with life expectance is meaningful. With such a broad definition of multimorbidity (any 2+ conditions from a list 36 diseases), the reduction in life expectancy is heavily affected by the specific distribution of the 36 conditions in this highly-selected study population. The finding is by no means generalisable. Thus, I would drop that from the synopsis of the main findings. The other main finding is that "not all lifestyle risk factors are equal" - this has long been known and has been well documented, so I suggest the authors also drop that point. In my opinion, the main finding of this study is that a heathy lifestyle is equally important in term of life expectancy for people with and without multimorbidity. This is a novel and surprising finding which the author should highlight more as it shows how important these factors are for the prognosis/outcome of multimorbidity.	In the revised first paragraph of the discussion, we have restructured the text highlighting the main finding (the effect of a healthy lifestyle is consistent across multimorbidity status) and toned down the other finding showing a dissimilar impact of each lifestyle on the risk of death (defined as "confirmatory results") [lines 359-370].<br><br>We have also focused more in the remaining discussion about the main finding [lines 425-433] and modified the "AUTHOR SUMMARY" box to reflect these suggestions.
9. Discussion, 2nd para. Here results from a supplementary analysis of unweighted lifestyle score (the sum of lifestyle risk factors) would allow a more direct comparison for other studies.	We have added this new analysis in the revised paper and compared the results with other studies that used unweighted lifestyle score [lines 378-380; 402-408].

Reviewer #2	
**Comments**	**Response**
10. Limitations section. A bit more discussion on generalisability is needed. The 5% response rate in the UK Biobank is exceptionally low by any standards. Selection has been shown to have affected disease prevalence in the cohort. But the key issue here is whether selection is likely to have affected associations between lifestyle and life expectancy. There are studies comparing risk factor-disease outcome associations in UK Biobank and studies with conventional response rates which could help to evaluate this.	Thank you for highlighting this point.<br><br>We have further expanded on the generalisability of UK Biobank [lines 442-452]. We have also quoted the very recent manuscript indicating that subjects need not to be representative for risk factor associations [Ref. 41], although the sample population is poorly generalisable [Ref. 38]. Furthermore, we discussed the implications of generalisability in terms of relative and absolute risk estimates [lines 444-452].
11. Several recent studies have examined lifestyle scores in relation to disease-free life expectancy and the results are well in agreement with the current figures on life expectancy - approximately 10+-2 years difference between people with the healthiest versus unhealthiest lifestyle factors (e.g. Zaninotto et al Sci Rep 2020, Nyberg et al JAMA Intern Med, Li et al BMJ 2020). The authors might consider highlighting this close agreement in results across health span and life span.	Thank you very much for quoting these three very recent papers.<br><br>One was actually included in our manuscript but we incorrectly reported the name instead of surname in the supplementary table S1 summarising previous evidence. The other two (Nyberg – JAMA Int Med. 2020;180(5):760-768; Zaninotto – Scientific Reports. 2020;10(1):1-9) have been published during the review process after our submission.<br><br>In the revised paper, we have added in S1 Table these two studies and mentioned the consistency of our results with these two studies [line 398-402]
12. Final paragraph of the discussion, the last sentence. It is well-known that risk factors are not equally strongly associated with life expectancy or mortality - highlighting this as a main conclusion makes this paper look quite non-innovative. I suggest dropping the last sentence.	Thanks again. We have removed the part related to risk factors.<br><br>Many thanks for your insightful suggestions and your time to review our manuscript.

<table>
<tr><th colspan="2">Reviewer #3</th></tr>
<tr><th>Comments</th><th>Response</th></tr>
<tr><td>In this study, the authors determined the effect of adherence to a healthy lifestyle on life expectancy in adults with and without known co-morbidities. Using the data from U.K. Biobank, the authors concluded that regardless of the presence of multimorbidity, engaging in a healthier lifestyle is associated with up to 8 years longer life. The study is of great public health importance.
I have the following comments and suggestions:

1. Given that these results are of great interest to public health, I suggest that scoring of adherence to a healthy lifestyle should be defined differently and simplified. For each lifestyle factor studied, the participants get a score of 1 if they met the healthy definition and 0 if they not. Then, sum these 4 scores and create an overall index of healthy lifestyle ranging from 0-4, with higher scores indicating a healthier lifestyle. The advantage of this simple score is that we get a sense of risk reduction or increased life expectancy if the population shifts to a healthier lifestyle (e.g., from 2 to 4 healthy lifestyle factors). Weighing the score is a sound method that the authors clearly explain in the Discussion, but does not align well with the public health interest.</td><td>Thank you.

We completely agree. We have added the analysis using the unweighted sum. We would prefer to keep both previous analysis and this suggested one to make our results and message more robust.

Results are reported in the “Sensitivity analyses” paragraph [lines 333-353] and shown in S13 Table (for multimorbidity 2+) and S14 (for cardiometabolic multimorbidity; see also next answer to next question). The years of life gained were slightly greater comparing heathiest vs unhealthiest groups in this analysis vs the analysis using the original weighted score [lines 348-351], but were on overall largely confirmed. Interestingly, using this approach our results could be more easily compared to other studies and are very well in line with some of the previous evidence using a similar approach [lines 378-380; 402-406].</td></tr>
<tr><td>2. The group with multimorbidity is very heterogeneous, while the group without co-morbidity is homogenous. The study population with multimorbidity included adults with less life-threatening conditions such as glaucoma, hypertension, sinusitis, rheumatoid arthritis, and those with diseases such as heart disease, stroke, cancer, dementia that are leading causes of death. I suggest authors create subgroups of people with multimorbidity and take into account the severity of the disease. Or, authors may apply a weighted score to account for the disease severity.</td><td>In line with another similar comment, we have added a sensitivity analysis using more homogenous definition of multimorbidity. We defined clusters of cardiometabolic conditions, which included diabetes + CVD (stroke, myocardial infarction, heart failure, angina or peripheral vascular disease). The results are reported in the “Results” paragraph [lines 340-348; 351-353] and in Table S14.

These analyses highlighted the very few number of participants and events (particularly in women) which hampered a straightforward comparisons with findings using multimorbidity defined as 2+ conditions, yet for the individual risk factors the results were qualitatively similar. Therefore, we highlighted in the discussion [lines 475-481] that further research is required to explore whether a lifestyle factors and a healthier lifestyle is differently associated with life expectancy in relation to the types of multimorbidity.

About the possibility to consider severity of the disease, in a previous investigation we showed that results are consistent when using simple frequencies of diseases or adding also self-reported overall health – which can be considered a proxy of disease severity. We have mentioned that in the discussion [lines 470-472; Ref. 8].</td></tr>
</table>

Reviewer #3	
**Comments**	**Response**
3. The authors compare people with and without multimorbidity regarding the effect of lifestyle factors and life expectancy, but how different are those groups concerning sample size, demographic, lifestyle, and other clinical factors? To have a fair comparison between groups, the authors should match people with and without multimorbidity and conduct the analysis.	Although matching provides a solution to account for differences between the groups being compared, we opted to adjust (rather than match) as there is no clear evidence of statistical advantages of matching over adjusting [Elze M et al. 10.1016/j.jacc.2016.10.060]. Moreover, matching would result in a reduction of the sample size compared to regression adjustment.<br>Key potential confounders of the association between lifestyle and mortality, namely ethnicity, socio-economic status, body mass index and employment, were accounted for in the adjusted survival analyses. The sample size difference are also accounted for and statistically expressed by the width of the confidence interval.
4. The authors report more than 50% of the study population as very healthy according to the weighted score. In the U.S., about 5% of the study population met the overall healthy lifestyle (Li et al. BMJ 2020; 368).	We believe that this is related to the representativeness of UK Biobank compared to Nurses' Health Study/Health Professionals Follow-Up Study in Li et al.<br><br>We have further expanded on the generalisability of UK Biobank [lines 442-452]. We have also quoted the very recent manuscript indicating that subjects need not to be representative for risk factor associations [Ref. 41], although the sample population is poorly generalisable [Ref. 38]. Furthermore, we discussed the implications of generalisability in terms of relative and absolute risk estimates [lines 444-452].<br><br>Many thanks for your helpful suggestions and your time to review our manuscript.
