# Supplementary material for: Healthy lifestyle and life expectancy in people with multimorbidity in the UK Biobank: A longitudinal cohort study
Source: PLoS Med. 2020 Sep 22;17(9):e1003332. doi: 10.1371/journal.pmed.1003332 (PMC7508366; doi:10.1371/journal.pmed.1003332)
Supplement: Attachment — Submitted filename: R2_Response to reviewers.pdf [file pmed.1003332.s028.pdf]

Requests from the editors	
**Comments**	**Response**
1. Please respond to the comments of reviewer #3, specifically relating to an additional matching/specificity analysis.	We have now carried out the requested additional matching analsyes according to reviewer #3; the results are consistent with those of the main analysis and are reported in S5 Figure and S15 Table.
2. Abstract:<br>a) Line 53: Given the high p value for this particular result, please rephrase the following to say that the association was not significant: "At 45 years, in men with multimorbidity an unhealthy score was associated with a gain of 1.5 (95% CI: -0.3, 3.3; P=0.572) additional life…"<br>b) In the last sentence of the Abstract Methods and Findings section, please describe the main limitation(s) of the study's methodology more clearly, and use the word "limitation(s)".<br>c) Line 65: Please replace "contributed to" with "correlated with".	We have amended the text has indicated.
3. Please remove spaces in your citation callouts, eg. "…mental health conditions [1,2],…"	The spaces have been removed.
4. Please move the Ethics statement from page 21 to the Methods section.	The statament has been removed from page 21 and reported only in the "Methods" section [lines 153-155].
5. Please move the Data statement from page 21 to the submission form.	We have moved the statement.
6. Please provide more access details (eg. URL, DOI, or issue/page nos.) for references 12, 16, and 34.	These details have been provided now.<br><br>Many thanks for your time to review our manuscript.

**Reviewer #3**	
**Comments**	**Response**
I thank the authors for responding to my comments.	Thank you.
1. I noted that when the analysis was focused on individuals with diabetes, heart disease and stroke, authors reported "imprecise HR and years of life gained estimates" due to the limited number of people with events. Does this indicate a presence of selection bias in the study? The authors are studying life expectancy, but the number of people with life-threatening diseases such as heart disease and stroke does not allow authors to conduct a rigorous analysis among this group? The authors may compare the prevalence of the cardiovascular disease in the UK biobank with national statistics in the UK.	Following the previous suggestions, we have limited the definition of multimorbidity to cardiometabolic conditions. This resulted in a reduction of the sample size compared to the definition including any 2+ chornic diseases: such reduction is of a great magnitude in the UK Biobank as this cohort is not fully representative of the general UK population.<br>This limitation has been mentioned in the manuscript, where we quoted two important previous studies investigating the implications of such limited representativness [ref. 38 & 41]. In fact, on the relative risk scale, the estimates are not biased if the cohort is not representative [ref. 41] while, on the absolute risk scale, the greater risk in the general population compared to that estimated in our study would result in a larger benefit of a healthy lifestyle in the genarel population [lines 448-455; ref. 40]<br>Therefore, we believe that the analysis is rigouorus (i.e., not biased) but the estimates may be imprecise because there not many events (i.e., the statistical power may be limited). This is indirectly highlighted by the width of the confidence intervals.<br>As suggested, in the revised text we have reported the prevalance of CVD in UK [Ref. 43; lines 481-485].
2. Concluding that the effect of lifestyle factors is similar in people with and without multimorbidity is simplified, in my opinion. First, there is no analysis to compare head-to-head the role of lifestyle factors on life expectancy between the groups with and without multimorbidity. The similarities or differences in life expectancy in each group could be explained by other factors and not necessarily to the adherence to a healthy lifestyle. For this reason, I recommended that authors conduct a matching analysis (as a sensitivity analysis), in which they will select people with multimorbidity with similar lifestyle scores and other characteristics (age, gender, BMI, social status) to people without multimorbidity. As a result of matching, they will create two "identical" groups in terms of sample size, lifestyle, and other characteristics, but different from the presence of multimorbidities. Then, in each group, separately, they will compare the role of	We have now conducted an analysis matching participants with multimorbidity to those without. The matching was performed with a propensity score approach using a single nearest-neighbour matching without caliper for all participants with multimorbidity.<br><br>The results of the matching procedure are shown in S5 Methods while the results of the survival analysis using the cohort of subjects without multimorbidity (74,013 participants) "matched" to those with multimorbidity are presented in the S5 Figure and S15 Table.<br><br>These results are largely consistent with those obtained from the non-matched cohort and, in some cases, the years of life gained are slightly greater.<br><br>We have mentioned in the text this new sensitivity analysis [Methods, line 270-272; Results, line 354-356]. Many thanks for this suggestion.

Reviewer #3	
**Comments**	**Response**
adherence to a healthy lifestyle in life expectancy.	
3. Second, there is no information about the severity of diseases and the role of medications in people with multimorbidities. It could be that those who adhere to a healthier lifestyle could have less severe conditions and proper adherence to medication, suggesting that these findings could be attributed to disease severity and treatment management, not lifestyle factors adherence.	We recognise that there are levels of severity for chronic conditions which unfortunately cannot be easily collected in a very large epidemiological study, in line with the well-known trade-off between phenotypical details and sample size. We mentioned in the limitation of the manuscript that in one of our previous investigation the results were consistent whether or not severity was accounted for in the definition of multimorbidity (Ref. 8 in the manuscript; lines 473-477).<br><br>Regarding specifically medication use, unfortunately we cannot test the correlation between healthy lifestyle and adherence to medications because in UK Biobank there are no assessments of adherence (i.e., from urine/blood samples).<br><br>In the revised manuscript, we have recognised that a healthier lifestyle may be also marker of a greater adherence to medications [lines 459-460].
4. Third, from the public health perspective, the guidelines that authors based on the lifestyle score (e.g., physical activity >150 minutes/week of moderate activity or 75 minutes of vigorous activity) are for primary prevention of chronic diseases such as cardiovascular disease (e.g., heart disease and stroke) and not secondary prevention. For example, according to the American Heart Association/American Stroke Association, physical activity recommendations for stroke survivors should be customized for each individual and should promote low- to moderate-intensity aerobic activity (Stroke. 2014;45:2532-2553).	Thank you for this comment. There are some differences between the US and UK guidelines regarding lifestyle interventions to reduce the risk of CVD in the general population, as well as specific guidelines may report different thresholds according to some patient's characteristics (i.e., age or sex), primary/secondary prevention, or type of CVD condition.<br><br>Therefore, in the amended manuscript, we have underlined [lines 498-499] that these guidelines are for the general population but personalised lifestyle programs should be considered for the single patient.<br><br>Many thanks for your time to review our manuscript and for your comments.
