# Supplementary material for: Healthy lifestyle and life expectancy in people with multimorbidity in the UK Biobank: A longitudinal cohort study
Source: PLoS Med. 2020 Sep 22;17(9):e1003332. doi: 10.1371/journal.pmed.1003332 (PMC7508366; doi:10.1371/journal.pmed.1003332)
Supplement: Attachment — Submitted filename: Request from editors & production.pdf [file pmed.1003332.s029.pdf]

Requests from the editors	
**Comments**	**Response**
1. Please update the Title to: "Healthy Lifestyle and Life Expectancy in people with Multimorbidity in the UK Biobank: a longitudinal cohort study"	Updated.
2. Abstract:<br>a. Line 43: Please use square brackets when nesting inside other brackets: "…(median age of 58 years [range 38-73], 46% male, 95% white)…"<br>b. At line 55, please adapt the text to "... significantly associated with a gain of 4.5 life years ...".<br>c. Please add another limitation to the end of the Methods and Findings subsection, eg. participants not being representative of the UK as a whole.<br>d. At line 64, please begin the sentence with "In this analysis of data from the UK Biobank, we found that ..." or similar.<br>e. Rather than "up to 8 years" at line 65, we suggest quoting the observed maxima for men and women (i.e., 6.3 and 7.6 years).	This has been adjusted as requested.
3. Methods: Please cite the prospective protocol on line 146 for clarity.	The protocol has now been cited.
4. Tables 2, 3: Please include p values for all relevant results.	P-values have been included in Tables 2 and 3.
5. Please upload the STROBE S1 Checklist as a separate file.	The STROBE checklist will be uploaded as a separate file.

Requests from Production	
**Comments**	**Response**
**FIGURES and GRAPHICS:**<br>1. Figures 1 and 2 exceed the dimension limits for figures (look at your Figures in Outline View to see how). Figures must be less than 19.05 cm in width and less than 22.225 cm in height (excluding white space around your artwork).<br><br>Please make the necessary adjustments and re-upload your figures.<br><br>-In Photoshop: Image→Image Size. Make sure the Resample Image checkbox is NOT checked. If the Width is over 19.05 cm, type 19.05 in the Width box (19.05 cm is our maximum allowable width for figures).<br>-In GIMP: Image→Scale Image. Set the units of measurement, in the pull down menu next to Height, to millimeters. If the Width is over 190.5 mm, type 190.5 in the Width box (19.05 cm is our maximum allowable width for figures) and hit Tab. The new Height of the figure will appear, scaled proportionately to the change in Width.	The figures have been resized.
**METADATA:**<br>1. Please ensure your short title is correct in the submission form, including symbols, italicisation etc. Your short title will be typeset from the submission form directly.<br><br>2. Please accurately complete the 'Competing Interests' section on our submission form, including any COIs declared by your co-authors. If you have no competing interests to declare, please add "The authors have declared that no competing interests exist". Otherwise please declare all competing interests beginning with the statement "I have read the journal's policy and the authors of this manuscript have the following competing interests:"<br><br>3. Your co-author, Dr. Yates, has declared the following competing interest in relation to this manuscript. Please include it in the main competing interest statement in our system, sticking as closely as possible to Dr. Yates's wording, using only their initials.<br><br>"Funding received from the Leicester NIHR Leicester BRC"<br><br>4. Your co-author, Dr. Davies, has declared the following competing interest in relation to this manuscript. Please include it in the main competing interest statement in our system, sticking as closely as possible to Dr. Davies's wording, using only their initials.<br><br>"Professor Melanie Davies has acted as consultant, advisory board member and speaker for Novo Nordisk, Sanofi-Aventis, Lilly, Merck Sharp & Dohme, Boehringer Ingelheim, AstraZeneca and Janssen, an advisory board member for Servier and Gilead Sciences Ltd and as a speaker for NAPP, Mitsubishi Tanabe Pharma Corporation and Takeda Pharmaceuticals International Inc."	The metadata has now been updated.

Requests from Production	
**Comments**	**Response**
5. In accordance with PLOS' data policy, please ensure that your Data Availability Statement in the submission form clearly identifies how readers can access your data. Note that it will be typeset as it is written so please ensure it is in complete sentences and appears as you would like it to in the published version.<br><br>For details on our data policy, please see https://eur03.safelinks.protection.outlook.com/?url=http%3A%2F%2Fjournals.plos.org%2Fplosmedicine%2Fs%2Fdata-availability&data=02%7C01%7Cyc244%40leicester.ac.uk%7C82901505346a4aac40df08d833a75ab8%7Caebecd6a31d44b0195ce8274afe853d9%7C0%7C0%7C637316141155008444&sdata=0Hht0x4stvWIw%2FHN9TGGHErhWTgWpestXiap41z0TlA%3D&reserved=0	
**ARTICLE FILE:**<br>1. Please indicate the corresponding author on the title page with an asterisk (*) and include the corresponding author's email address. Only an asterisk and the email address itself are required.<br><br>2. Please remove author degree titles from the byline of your manuscript file.<br><br>3. Please ensure that your Ethics Statement is available in the Methods section of your manuscript in its entirety.<br><br>4. We have noted that you have the following present in your manuscript: Abbreviations list<br>We have removed these section from your manuscript file as this information is automatically pulled from the submission form. Please make sure that the information in the submission form is correct.	This has been ajusted as requested.
**ACKNOWLEDGMENTS:**<br>1. Please remove the funding information from the Acknowledgments section of your manuscript file. The published version is pulled automatically from the “Financial Disclosure” field in our submission form and must not be duplicated. Please therefore take this opportunity to check that the information in this field is correct and completed in full.<br><br>2. Please move the NIHR/Department of Health and Social Care disclaimer to the end of the Acknowledgments section of your manuscript, with a line break between the main text of the acknowledgments and the disclaimer statement.	The funding information has been removed and a line break added.

Requests from Production	
**Comments**	**Response**
**REFERENCES:**<br>1. Please make sure all references are cited in ascending numerical order in the text.	Re-checked.
**SUPPORTING INFORMATION:**<br>1. Please upload your Supporting Information items individually, rather than in a single file, as you refer to them individually in the text. Supporting Information files should be less than 10 MB each, and the individual figure files should be labelled clearly eg “S1 Fig”, "S1A Fig" etc.<br><br>2. Please ensure that all Supporting Information files are clearly labeled in the file inventory. i.e. S1_Fig.tif, S1_Table.xlsx, S1_Text.doc, S1_Data.xlsx, S1_PRISMA_Checklist.docx, etc.<br><br>Each file type should be numbered starting at 1. Please also make sure that the file description matches the file name (e.g. S1_Fig.tif is listed as S1 Fig).<br><br>3. Please ensure that each supporting information file has a legend at the end of the manuscript file, after the Reference list.<br><br>4. Please note that your submission cannot contain any references to generic "supporting information" files and instead must refer to specific files using our naming convention (S1 Fig, S1 Table, S1 Text, S1 Data, etc.)	Files have been modified as requested and will be uploaded separately.
To opt in or out of publishing the peer review history for this article.	Opt in.<br>We would like to publish the peer review history for this article.
